# Patterns of childhood body mass index (BMI), overweight and obesity in South Asian and black participants in the English National child measurement programme: effect of applying BMI adjustments standardising for ethnic differences in BMI-body fatness associations

**DOI:** 10.1038/ijo.2017.272

**Published:** 2018-02-06

**Authors:** M T Hudda, C M Nightingale, A S Donin, C G Owen, A R Rudnicka, J C K Wells, H Rutter, D G Cook, P H Whincup

**Affiliations:** 1 Population Health Research Institute, St George’s, University of London, London, UK; 2 Childhood Nutrition Research Centre, Population, Policy and Practice Programme, UCL Great Ormond Street Institute of Child Health, London, UK; 3 ECOHOST—The Centre for Health and Social Change, London School of Hygiene and Tropical Medicine, London, UK

## Abstract

**Background:**

The National Child Measurement Programme (NCMP) records weight and height and assesses overweight-obesity patterns in English children using body mass index (BMI), which tends to underestimate body fatness in South Asian children and overestimate body fatness in Black children of presumed African ethnicity. Using BMI adjustments to ensure that adjusted BMI was similarly related to body fatness in South Asian, Black and White children, we reassessed population overweight and obesity patterns in these ethnic groups in NCMP.

**Methods:**

Analyses were based on 2012–2013 NCMP data in 582 899 children aged 4–5 years and 485 362 children aged 10–11 years. Standard centile-based approaches defined weight status in each age group before and after applying BMI adjustments for English South Asian and Black children derived from previous studies using the deuterium dilution method.

**Findings:**

Among White children, overweight-obesity prevalences (boys, girls) were 23% and 21%, respectively, in 4–5 year olds and 33% and 30%, respectively, in 10–11 year olds. Before adjustment, South Asian children had lower overweight-obesity prevalences at 4–5 years (19%, 19%) and slightly higher prevalences at 10–11 years (42%, 34%), whereas Black children had higher overweight-obesity prevalences both at 4–5 years (31%, 29%) and 10–11 years (42%, 45%). Following adjustment, overweight-obesity prevalences were markedly higher in South Asian children both at 4–5 years (39%, 35%) and at 10–11 years (52%, 44%), whereas Black children had lower prevalences at 4–5 years (11%, 12%); at 10–11 years, prevalences were slightly lower in boys (32%) but higher in girls (35%).

**Interpretation:**

BMI adjustments revealed extremely high overweight-obesity prevalences among South Asian children in England, which were not apparent in unadjusted data. In contrast, after adjustment, Black children had lower overweight-obesity prevalences except among older girls.

**Funding:**

British Heart Foundation, NIHR CLAHRC (South London), NIHR CLAHRC (North Thames).

## Introduction

Childhood obesity is a major public health problem both globally^1^ and in England, where approximately one-third of children aged 2–15 years were recently reported to be overweight or obese using body mass index (BMI).^2^ Childhood overweight-obesity is associated with adult overweight-obesity,^3^ and with higher risks of type 2 diabetes and cardiovascular disease.^4, 5, 6^ Overweight-obesity in English South Asian and Black children of African origin is of particular concern; both ethnic groups have high type 2 diabetes and cardiovascular disease risks in adulthood,^7, 8, 9, 10^ originating in childhood.^11, 12^

Accurate assessment of overweight-obesity prevalence in English South Asian and Black children is therefore important. Most national surveys, including the National Child Measurement Programme (NCMP) and the Health Survey for England,^2, 13^ use BMI to categorise overweight and obesity identically in all ethnic groups. However, the relations between BMI and body fatness differ by ethnicity both in adults and children. Asian adults tend to have a lower BMI for a given body fatness than Whites.^14^ Among English children, more specifically, BMI systematically underestimates body fatness in South Asians and overestimates it in Blacks.^15, 16^ We recently developed ethnic-specific BMI adjustments, which provide adjusted BMI values for South Asian and Black children, which have the same relation to body fatness as in White children.^17^ In this report, we have applied these BMI adjustments to recent NCMP data to obtain an improved picture of the burdens of body fatness, as reflected in adjusted overweight-obesity prevalences in South Asian and Black children and in the English child population as a whole.

## Methods

### National Child Measurement Programme (NCMP)

#### Participants

The NCMP is an annual survey of the weights and heights of English children aged 4–5 years (reception year) and 10–11 years (Year 6) carried out since 2006–2007, currently directed by Public Health England; data collection is conducted by Local Authority (LA) public health departments.^18^ All state primary schools in England (*n*~17 000) are invited to participate; within participating schools, all relevant pupils are invited to participate on an opt-out basis. This report is based on the 2012/13 survey, the most recent for which relevant information including pupil ethnicity was available from the HSCIC (Health and Social Care Information Centre), now NHS Digital. Overall, 93% of eligible children participated.^13^

#### Data collection

Weight and height were measured by assessment teams recruited, trained and supervised by LA public health departments. Public Health England provided detailed instructions on instrument choice and calibration (requiring the use of annually checked Class III weight scales) and measurements, made without shoes in light indoor clothing. Weight was measured to the nearest 0.1 kg and height with the child’s heels together and the head in the Frankfurt plane to the nearest 0.1cm. BMI was calculated as weight per height^2^. School record information on name, date of birth, sex and parentally defined ethnicity was collected. Data were entered using the NCMP IT system and collated by HSCIC.

#### NCMP BMI category definitions

The NCMP uses the British 1990 child growth reference population (UK90) to assign each child a BMI centile taking into account their height, weight, sex and age.^18, 19^ Children are classified using population level thresholds as underweight (2nd centile or below), healthy weight (above 2nd centile, below 85th centile), overweight (on or above the 85th centile and below the 95th centile), or obese (on or above the 95th centile). ‘Overweight-obesity’ combines children who are overweight or obese (on or above the 85th centile). These population level thresholds follow standard NCMP reporting practice.^18^ More extreme clinical BMI centile thresholds identify children who are overweight (on or above the 91st centile, up to the 98th centile) or obese (on or above the 98th centile) as a basis for informing parents of their child’s weight status.^18^ Clinical ‘overweight-obesity’ refers to children on or above the 91st BMI centile.

#### Ethnicity

Ethnicity was defined using the National Health Service classification.^20^ For the present analyses, children identified as ‘White British’, ‘White Irish’ and ‘any other White background’ were grouped as ‘White’. Children identified as ‘Black African’, ‘Black Caribbean’ or ‘any other Black background’ were of presumed African origin and grouped together as ‘Black’. Children of ‘Indian’, ‘Pakistani’ or ‘Bangladeshi’ origin were grouped as ‘South Asian’. Children of ‘Chinese’ or ‘Asian other’ origins were grouped as ‘Other Asian’. Children of ‘any other ethnic background’ and ‘mixed ethnicity’ were grouped as ‘Other ethnicity’. Children with missing ethnicity data formed a separate category of 'Unknown'.

#### Adjusted BMI values for black and South Asian children

Ethnic-specific BMI adjustments for Black and South Asian children were derived using pooled data from four recent studies that used the deuterium dilution reference method to assess fat-free mass (and indirectly fat mass) in Black, South Asian and White children aged 4–12 years.^17^ BMI adjustments were derived using sex-stratified regression models, which ensured that adjusted BMI values were associated with fat mass (based on the reference method and expressed as a height independent fat mass index (fat mass per height^5^)) in the same way as in Whites.^17^ Regression models were adjusted for ethnic group and age group (in 3-year age groups (4.0–6.9, 7.0–9.9 and 10.0–12.9 years)) to provide robust and stable estimates. Model building was conducted using a stepwise forwards approach; two-way interaction terms between FMI, ethnic group and age group were included in the model and three way interactions were only considered if their corresponding two-way interactions were statistically significant at the 5% significance level.^17^ For South Asian children, single sex-specific-positive BMI adjustments of +1.12 kg m^−2^ for boys and +1.07 kg m^−2^ for girls were applicable for all age groups and body fatness levels. For Black children, negative BMI adjustments were needed which were modified by age and body fatness (Supplementary Table 1). Fuller details are provided in a previous report.^17^

### Statistical analysis

The distributions of weight, height and BMI were reviewed for outliers. BMI was positively skewed and therefore medians rather than means were presented. Median BMI and the prevalences of specific BMI categories (underweight, normal weight, overweight, obese) defined using the UK90^19^ were determined for each ethnic group and for all participants before and after the application of BMI adjustments. Mann–Whitney *U*-tests were used to compare the distributions of BMI (or adjusted BMI) and indirectly to compare the differences in medians; *z*-tests for differences in proportions were used to compare prevalences of overweight-obesity between each of the ethnic minority groups and the White children. The prevalence of overweight-obesity was also determined for each LA in England to allow geographical comparisons to be made (including both prevalence and prevalence rankings) before and after BMI adjustment.

### Role of the funding source

The funder had no role in the study design, data analysis, data interpretation or writing of the report. The authors had full access to all the data in the study and had final responsibility to submit the manuscript for publication.

## Results

### Participants and data exclusions

In the 2012–13 school year, 1 076 824 children participated in NCMP. Of these, we excluded 8563 children (0.01%) from analyses. Four children had implausible weight or height values and 324 children were outside the study age-range. A further 8235 children who were measured in LAs identified by NCMP as having data quality concerns (Redcar-Cleveland, Torbay and Middlesbrough) were excluded. Children from one further area (Bassetlaw) flagged up by NCMP for potential data quality concerns were not excluded; Bassetlaw was part of a substantially larger LA district (Nottinghamshire) without quality concerns. LA analyses specifically excluded 684 children from three LAs each with fewer than 1000 participants (The City and County of the City of London (*n*=11), Isles of Scilly (*n*=21) and Rutland (*n*=652)) to avoid unnecessary imprecision in the results.

### Characteristics of study participants

Table 1 summarises participant characteristics for each age–sex group, including 582 899 children aged 4–5 years and 485 362 children aged 10–11 years from 152 LAs. Ethnicity prevalences (~60% Whites, ~5% Blacks, ~8% South Asians) did not differ appreciably by age–sex group. Data on ethnicity were not available for ~13% of 4–5 year olds and ~16% of 10–11 year olds. As expected, older children were heavier and taller on average and had higher median BMIs. At 4–5 years, boys were heavier and taller than girls, with a marginally higher median BMI; at 10–11 years, girls were heavier and taller than boys and had a higher median BMI.

### Median BMI and prevalences of overweight, obesity and overweight-obesity by ethnicity: effect of BMI adjustments

Median BMI and prevalences of BMI categories by ethnicity before and after BMI adjustment are shown for 4–5 year olds in Tables 2 and 3 and for 10–11 year olds in Tables 4 and 5. In White children, the prevalences of overweight-obesity (boys, girls) were 23.0% and 20.9% in 4–5 year olds, 32.8% and 30.4% in 10–11 year olds, respectively.

#### Black children

Before BMI adjustment, Black children had higher median BMI than Whites for all age–sex groups (Mann–Whitney *U*-tests, all *P*<0.0001). The prevalences of overweight-obesity and obesity were higher than those of White children, both for boys and girls at 4–5 years and at 10–11 years (*z*-tests, all *P*<0.0001). However, after adjustment, Black children aged 4–5 years (both boys and girls) and 10–11-year-old boys had slightly lower median adjusted BMI whilst Black 10–11-year-old girls had higher adjusted BMI (compared with Whites) (Mann–Whitney *U*-tests, all *P*<0.0001). Overweight-obesity prevalences were slightly lower in Black children aged 4–5 years (both boys and girls) (*z*-tests, both *P*<0.0001) and in 10–11-year-old boys (*z*-test, *P*=0.04). However, black girls aged 10–11 years had a higher overweight-obesity prevalence than their White peers (*z*-test, *P*<0.0001). There were no consistent differences in median adjusted BMI and overweight-obesity prevalence between Black African, Black Caribbean and other Black children either before or after adjustment.

#### South Asian children

before BMI adjustment, BMI patterns in South Asian children differed by age group. At 4–5 years, median BMI was lower in South Asians than in White children (Mann–Whitney *U*-tests, both *P*<0.0001). Overweight-obesity prevalences were also lower in 4–5-year-old South Asians than in White children (*z*-tests, both *P*<0.0001). At 10–11 years, South Asian boys had an appreciably higher median BMI than Whites (Mann–Whitney *U*-test, *P*<0.0001) but there was no marked difference in girls (Mann–Whitney *U*-test, *P*=0.77). However, overweight-obesity prevalences for both boys and girls were higher than White children (*z*-test, both *P*<0.0001). After adjustment, South Asian children (boys and girls), both at 4–5 years and more so at 10–11 years, had higher median BMIs (Mann–Whitney *U*-tests, all *P*<0.0001); they also had higher overweight-obesity prevalences than White children (*z*-tests, all *P*<0.0001); more than half of older South Asian boys were overweight-obese. Within the South Asian group, children of Pakistani and Bangladeshi origin had higher median adjusted BMI, obesity and overweight-obesity prevalences than children of Indian origin; these patterns were observed for both boys and girls.

### Prevalences of underweight and healthy weight by ethnicity: effect of BMI adjustments

Unadjusted underweight prevalences were higher in younger Black children and similar in older Black children compared with Whites; South Asian children had higher unadjusted prevalences of underweight in both age groups. However, after adjustment Black children had even higher, and South Asian children lower, underweight prevalences. Unadjusted healthy weight prevalences were lower in Black children (younger and older) compared with Whites; younger South Asian children had similar unadjusted healthy weight prevalences to Whites, while older children had lower prevalences. However, adjusted prevalences of healthy weight were markedly higher in Black children and markedly lower in South Asian children compared with White children.

### Overall median BMI and prevalences of weight categories: effect of BMI adjustments

The effects of ethnic-specific BMI adjustments on overall BMI and overweight-obesity patterns in the NCMP population were also examined (Table 2–5). After BMI adjustment, overall population median BMI values and the prevalences of being underweight or healthy changed very little. The adjusted overall prevalences of overweight-obesity were marginally increased in all age–sex groups, all by 0.5% or less.

### LA differences in overall overweight-obesity prevalence: effect of BMI adjustments

**Figure 1:**
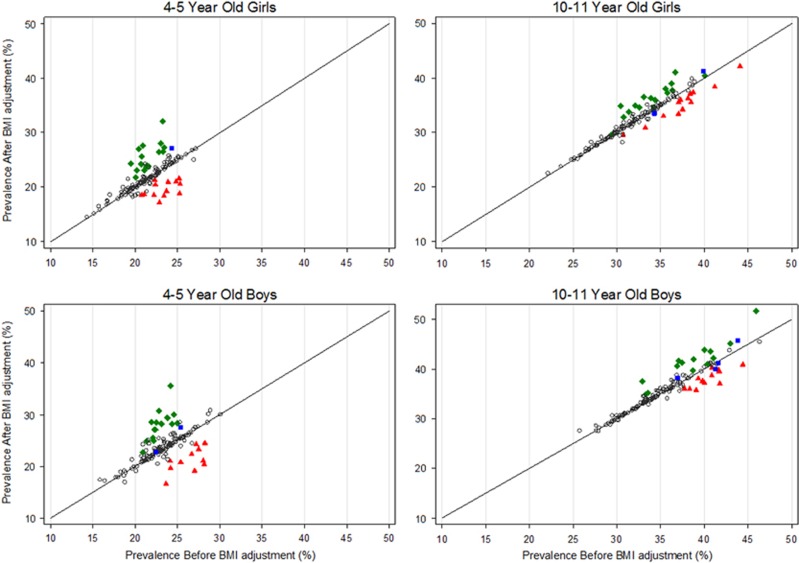
Correlation of prevalences of overweight-obesity in local authorities before and after BMI adjustments by age–sex group in the national child measurement programme (2012–13). Local authorities are colour coded by ethnic composition. Legend: open circles=South Asian & Blacks<20%, green diamond=South Asian⩾20% & Blacks<20%, red triangle=Blacks⩾20% & South Asian<20%, blue square=South Asian & Blacks⩾20% Based on the overweight-obesity population thresholds: overweight-obese: ⩾85th centile Excluding areas with potential data quality issues and areas with <1000 individuals.

The effects of BMI adjustment on the prevalences and rankings of overweight-obesity in LA areas were examined. Prevalences of overweight-obesity in LA areas before and after BMI adjustments are plotted against one another in Figure 1, for each age–sex group. LA variations in overweight-obesity prevalence were marked in 10–11 year olds (20–50%); the Spearman rank correlations of unadjusted and adjusted prevalence were high both for boys and girls (both *ρ*=0.96). LA variations in overweight-obesity prevalence were smaller in 4–5-year-old boys and girls (15–30%) and correlations between unadjusted and adjusted prevalence were weaker (*r*=0.62, 0.74, respectively). After adjustment, overweight-obesity prevalences in LAs with a high South Asian population prevalence (⩾20%) were systematically higher, whereas prevalences in LAs with a high Black population prevalence (⩾20%) were systematically lower. In the small number of LAs with a high population prevalence of both ethnicities, adjustment had little effect on overweight-obesity prevalences (Figure 1). However, the effects of BMI adjustment on LA rankings were substantial. The 20 LAs with the highest overweight-obesity prevalences both before and after BMI adjustment in each age–sex group are summarised in supplementary Figures 1–4. In 4–5 year olds, more than half of the 20 LAs with high overweight-obesity rankings were different after BMI adjustment; in 10–11 year olds, at least a quarter were different. After adjustment, more LA areas with a high South Asian population prevalence were present in the top 20 rankings, whereas the number of LA areas with a high Black population prevalence declined (Supplementary Figures 1–4). A complete summary of LA overweight-obesity prevalences before and after BMI adjustment for each age–sex group is presented in Supplementary Table 2; corresponding information on overweight-obesity prevalence rankings is presented in Supplementary Table 3.

### Sensitivity analyses

To determine whether results were influenced by children with particularly high unadjusted BMI values, sensitivity analyses excluded children with severe obesity (*n*=14 087), defined using age and sex-specific Extended International Obesity Task Force thresholds.^21^ The results were not materially affected by excluding these individuals. The results were also examined using more extreme overweight-obesity definitions, those based on the use of NCMP clinical reporting thresholds (on or above the 91st percentile). The patterns of ethnic differences in overweight-obesity prevalence were not materially changed by the use of more extreme thresholds (Supplementary Table 4).

## Discussion

In this study, the first to our knowledge using ethnic-specific BMI adjustments to obtain an accurate picture of the relative prevalences of overweight-obesity in English children of different ethnicity, adjusted childhood overweight-obesity prevalence was particularly high among South Asian children in all age–sex groups and among older Black girls. These patterns were markedly different from those based on unadjusted BMI data, in which higher overweight-obesity prevalences in Black children were apparent. BMI adjustment increased the prevalences and rankings of overweight-obesity in LAs with high South Asian representation (⩾20%) and reduced them in LAs with high Black representation.

### Relation to previous studies

In the present investigation, unadjusted median BMI and overweight-obesity prevalences were particularly high in Black children compared with Whites, both at 4–5 years and at 10–11 years. This is consistent with previous NCMP reports from the same^13^ and previous years,^22^ and with BMI data from other nationally representative studies, including the Health Survey for England^7^ and the Millennium Cohort Study both at 5 years^23^ and 11 years.^24^ The unadjusted BMI patterns in South Asian children, with lower unadjusted median BMI and overweight-obesity prevalences than Whites at 4–5 years but higher prevalences at 10–11 years, are also consistent with NCMP data from the same^13^ and previous years^22^ and with reports from the Millennium Cohort Study.^23, 24^ The markedly higher adjusted median BMI and overweight-obesity prevalences levels observed among South Asian children at both 4–5 and 10–11 years are consistent with the results of other population-based studies using more direct body fatness measures, including bioimpedance and skinfold thickness in 9–10 year olds,^15^ deuterium dilution in both 8–10 year olds^16^ and 5–11 year olds^25^ and dual energy X-ray absorptiometry in 5–18 year olds,^26^ all of which showed higher body fatness in South Asians than in Whites. The lower adjusted median BMI and overweight-obesity prevalences observed in all Black children (except older girls) are also consistent with the results of earlier studies using more direct body fatness measures, including bioimpedance and skinfold thickness in 9–10 year olds,^15^ deuterium dilution in 8–10 year olds^16^ and dual energy X-ray absorptiometry in 5–18 year olds,^26^ which all showed lower body fatness in Blacks than in Whites. Our results reinforce the conclusion of an earlier systematic review that observed ethnic patterns of childhood overweight-obesity are strongly dependent on the method used to assess overweight-obesity.^27^

### Implications

Our results, based on adjustment of BMI values to achieve consistent BMI-body fatness associations in South Asian, Black and White children, provide strong evidence that English South Asian children (especially Bangladeshis and Pakistanis) have elevated overweight-obesity burdens. This is a particular concern, given the high long-term risks of type 2 diabetes and cardiovascular disease in UK South Asians^7, 8^ from childhood.^11, 12^ A second concern is the high adjusted BMI values in older Black girls, which suggest that the patterning of high obesity prevalence in UK Black women^7^ is emerging between 4–5 and 10–11 years, again with implications for the focus of prevention in young age groups. The average differences in adjusted BMI of >1 kg m^−2^ (for example between South Asians and Whites at 10–11 years) would (if sustained into adulthood, which appears likely on current trends) account for appreciably higher risks of both T2D (by at least 25%)^28^ and CHD (by at least 5%);^5, 29^ the impact of higher BMI from childhood on T2D risk is likely to be particularly marked.^6^ The results also reinforce earlier concerns that unadjusted BMI data may disproportionately misclassify weight status in South Asian and Black children.^14, 15, 16, 25^ This report emphasises the scale of potential misclassification, showing that while unadjusted BMI data point to an excess of overweight-obesity in Black children, in reality the excess is greater in South Asian children—though overall overweight-obesity prevalences in the entire population of England are little affected, as the changes in South Asian and Black children tend to offset one another. The results also draw attention to uncertainties in overweight-obesity prevalence estimates at LA level, which have been reported annually by NCMP.^13^ These LA prevalence estimates are very sensitive to BMI adjustments and are particularly (and predictably) affected in LAs with high ethnic minority prevalences. Adjustment reduced overweight-obesity prevalence rankings in LAs with substantial Black representation and increased them in LAs with substantial South Asian populations. This underscores the need to treat LA rankings cautiously, and emphasise instead the widespread occurrence of childhood overweight-obesity in all English LAs; even among the lowest ranking LAs, overweight-obesity prevalences are excessive. Effective population-wide strategies for overweight-obesity prevention are therefore needed in all children, with a special emphasis on South Asian children and older Black girls. Although the present analyses focus on English children, the results are likely to be relevant for the UK as a whole. Moreover, they are likely to have relevance for other countries with substantial South Asian and African origin ethnic minority populations and could also have relevance for other ethnic minority populations (for example, Pacific Island populations) with different BMI-body fatness associations from those of majority White populations.^30, 31^

### Strengths and limitations

The NCMP is a large-scale, national survey resource with high rates of participation both by schools and individual children, with standardised data collection and quality control procedures. We used 2012–13 data, the latest year available to us. The validity of the BMI adjustments used is critical; these used the reference deuterium dilution method^32^ to obtain fat mass estimates based on a pooled resource of ~1750 Black, South Asian and White children. The BMI distributions of the South Asian, Black and White children in the studies used to derive BMI adjustments were very similar to those of the children in NCMP populations, suggesting that their application to NCMP data was appropriate. BMI adjustments were provided for South Asian and Black children (based on inclusion of Indian, Pakistani, Bangladeshi, Black African and Caribbean children); these groups together account for almost two-thirds of all ethnic minority participants in the NCMP. However, it was not possible to provide adjustments for other ethnic groups not represented in the deuterium studies, including children with mixed ethnicities. It is however possible that the adjustments derived for South Asian children could be applied to Other Asians,^14^ which would increase their estimated overweight-obesity burden. The validity of BMI adjustments could be greater if they could be standardised in relation to visceral fat (rather than total body fat), which is particularly implicated in the development of insulin resistance and type 2 diabetes risk and may be particularly high in South Asians.^8^ Although the validity and practicability of such adjustments remains uncertain, the current adjustments for South Asians may be conservative, potentially underestimating their true burden of overweight-obesity.

## Conclusion

There is a substantial excess of overweight-obesity among English South Asian children (both at 4–5 years and especially at 10–11 years) and among Black girls aged 10–11 years, with important implications for overweight-obesity prevention. These patterns are not apparent using unadjusted BMI data, which tend to underestimate overweight-obesity prevalences in South Asian children and overestimate them in Black children.

## Figures and Tables

**Table 1:** Characteristics of participants in the national child measurement programme (2012–13): by age–sex group

*Variable*	*4–5 Year olds* N=*582 899*		*10–11 Year olds* N=*485 362*	
	*Boys* N=*297 887*	*Girls* N=*285 012*	*Boys* N=*248 783*	*Girls* N=*236 579*
Age (years), mean (s.d.)	5.0 (0.3)	5.0 (0.3)	10.9 (0.4)	10.9 (0.4)
Height (cm), mean (s.d.)	110.0 (5.1)	109.0 (5.1)	144.7 (7.0)	145.4 (7.5)
Weight (kg), median (LQ–UQ)	19.3 (17.7–21.1)	18.9 (17.3–20.8)	37.5 (32.9–44.1)	39.0 (33.5–46.2)
BMI (kg m^−2^), median(LQ–UQ)	16.0 (15.2–16.9)	15.9 (15.1–17.0)	17.9 (16.3–20.4)	18.3 (16.5–21.0)
				
*Variable,*n*(%)*				
Ethnicity				
White	197 691 (66.4)	188 663 (66.2)	160 278 (64.4)	151 146 (64.0)
Black	14 468 (4.9)	13 970 (4.9)	11347 (4.6)	11 319 (4.8)
Black African	9003 (3.0)	8520 (3.0)	6172 (2.5)	6154 (2.6)
Black Caribbean	2838 (1.0)	2788 (1.0)	2971 (1.2)	2927 (1.2)
Black–Other	2627 (0.9)	2662 (0.9)	2204 (0.9)	2238 (1.0)
South Asian	23 191 (7.8)	22 109 (7.8)	19 406 (7.8)	18 636 (7.9)
South Asian–Indian	7474 (2.5)	7200 (2.5)	5817 (2.3)	5493 (2.3)
South Asian–Pakistani	11 502 (3.9)	10 855 (3.8)	9675 (3.9)	9322 (3.9)
South Asian–Bangladeshi	4215 (1.4)	4054 (1.4)	3914 (1.6)	3821 (1.6)
Other Asian	6184 (2.1)	6062 (2.1)	4325 (1.7)	41 745(1.8)
Other ethnicity	18 832 (6.3)	18 168 (6.4)	12 914 (5.2)	12 547 (5.3)
Unknown	37 521 (12.6)	36 040 (12.7)	40 513 (16.3)	38 756 (16.4)
				
*Region*				
East Midlands	25 212 (8.5)	23 837 (8.4)	21 496 (8.6)	20 232 (8.6)
East of England	34 041 (11.4)	32 391 (11.4)	28 195 (11.3)	26 143 (11.1)
London	47 613 (16.0)	46 331 (16.3)	37 346 (15.0)	36 701 (15.5)
North East	12 603 (4.2)	12 509 (4.4)	10 856 (4.4)	10 432 (4.4)
North West	40 997 (13.8)	38 947 (13.7)	34 734 (14.0)	32 848 (13.9)
South East	45 948 (15.4)	43 628 (15.3)	38 988 (15.7)	37 052 (15.7)
South West	26 481 (8.9)	25 377 (8.9)	22 370 (9.0)	21 136 (8.9)
West Midlands	33 353 (11.2)	31 636 (11.1)	28 430 (11.4)	26 999 (11.4)
Yorkshire and The Humber	30 590 (10.3)	29 206 (10.3)	25 026 (10.1)	23 696 (10.0)
Unknown	1049 (0.4)	1150 (0.4)	1342 (0.5)	1340 (0.6)

Abbreviations: LQ, lower quartile; UQ, upper quartile.

**Table 5:** 10–11-year-old girls–summary of body mass index and weight categories using UK90 population thresholds^a^ by ethnic group before and after ethnic adjustments to BMI in the national child measurement programme (2012–13)

*Ethnic group*	N	*Median BMI, Kg *m^*−2*^*(LQ–UQ)*	*10–11 -year-old girls prevalence, % (95 CI)*				Overweight-obese
			*Underweight*	*Healthy*	*Overweight*	*Obese*	
*Before BMI adjustment*							
White	151 146	18.3 (16.5–20.8)	1.2 (1.1–1.3)	68.4 (68.1–68.6)	14.2 (14.0–14.4)	16.2 (16.0–16.4)	30.4 (30.2–30.7)
Black	11 319	19.6 (17.3–22.7)	1.3 (1.1–1.5)	54.1 (53.2–55.0)	17.2 (16.5–17.9)	27.4 (26.6–28.2)	44.6 (43.7–45.5)
Black African	6154	19.6 (17.3–22.7)	1.4 (1.1–1.7)	53.5 (52.3–54.8)	17.5 (16.6–18.5)	27.5 (26.4–28.6)	45.0 (43.8–46.3)
Black Caribbean	2927	19.6 (17.2–22.8)	1.0 (0.6–1.3)	54.2 (52.4–56.0)	16.3 (14.9–17.6)	28.6 (27.0–30.2)	44.9 (43.1–46.7)
Black–Other	2238	19.4 (17.0–22.3)	1.4 (0.9–1.9)	55.5 (53.5–57.6)	17.5 (15.9–19.1)	25.6 (23.8–27.4)	43.1 (41.0–45.1)
South Asian	18 636	18.4 (16.1–21.4)	4.1 (3.8–4.4)	61.7 (61.0–62.4)	14.7 (14.2–15.2)	19.6 (19.0–20.1)	34.2 (33.5–34.9)
South Asian–Indian	5493	18.0 (15.9–20.9)	5.2 (4.6–5.7)	64.4 (63.1–65.7)	14.3 (13.4–15.3)	16.1 (15.1–17.1)	30.4 (29.2–31.7)
South Asian–Pakistani	9322	18.6 (16.2–21.6)	4.2 (3.8–4.6)	60.3 (59.3–61.3)	14.7 (14.0–15.4)	20.7 (19.9–21.5)	35.4 (34.5–36.4)
South Asian–Bangladeshi	3821	18.7 (16.4–21.8)	2.2 (0.5–0.8)	61.1 (59.5–62.6)	15.0 (13.9–16.1)	21.7 (20.4–23.1)	36.7 (35.2–38.3)
Other Asian	41 745	18.2 (16.2–20.6)	2.7 (2.2–3.2)	68.3 (66.9–69.7)	14.1 (13.1–15.2)	14.8 (13.8–15.9)	29.0 (27.6–30.3)
Other ethnicity	12 547	18.7 (16.6–21.6)	1.7 (1.5–2.0)	62.5 (61.7–63.4)	14.9 (14.3–15.5)	20.8 (20.1–21.5)	35.7 (34.9–36.6)
Unknown	38 756	18.3 (16.5–20.9)	1.5 (1.3–1.6)	67.4 (67.0–67.9)	14.2 (13.8–14.5)	16.9 (16.6–17.3)	31.1 (30.7–31.6)
Overall	236 579	18.3 (16.5–21.0)	1.5 (1.5–1.6)	66.7 (66.5–66.9)	14.4 (14.3–14.6)	17.3 (17.2–17.5)	31.8 (31.6–32.0)
							
*After BMI adjustment*^b^							
White							
Black		18.6 (16.6–21.4)	2.2 (1.9–2.5)	63.0 (62.1–63.9)	15.7 (15.0–16.4)	19.1 (18.4–19.8)	34.8 (33.9–35.7)
Black African		18.7 (16.6–21.4)	2.3 (1.9–2.7)	62.6 (61.4–63.8)	16.0 (15.0–16.9)	19.1 (18.1–20.1)	35.1 (33.9–36.2)
Black Caribbean		18.6 (16.5–21.5)	1.7 (1.2–2.2)	62.7 (61.0–64.5)	15.4 (14.1–16.7)	20.2 (18.7–21.6)	35.6 (33.8–37.3)
Black–Other		18.4 (16.4–21.1)	2.4 (1.8–3.0)	64.5 (62.5–66.5)	15.4 (13.9–16.9)	17.7 (16.1–19.3)	33.1 (31.1–35.0)
South Asian		19.5 (17.2–22.5)	0.6 (0.5–0.7)	55.8 (55.1–56.5)	17.3 (16.7–17.8)	26.4 (25.7–27.0)	43.6 (42.9–44.3)
South Asian–Indian		19.1 (17.0–21.9)	0.8 (0.5–1.0)	59.6 (58.3–60.9)	17.1 (16.1–18.1)	22.6 (21.5–23.7)	39.7 (38.4–41.0)
South Asian–Pakistani		19.6 (17.2–22.6)	0.7 (0.5–0.8)	54.7 (53.7–55.7)	17.1 (16.4–17.9)	27.5 (26.6–28.4)	44.7 (43.7–45.7)
South Asian–Bangladeshi		19.8 (17.5–22.9)	0.3 (0.2–0.5)	53.0 (51.4–54.6)	17.7 (16.5–19.0)	28.9 (27.5–30.4)	46.7 (45.1–48.2)
Other Asian							
Other ethnicity							
Unknown							
Overall		18.4 (16.5–21.1)	1.3 (1.3–1.3)	66.7 (66.5–66.8)	14.6 (14.4–14.7)	17.5 (17.3–17.6)	32.0 (31.9–32.2)

a Population thresholds: underweight: ⩽2nd centile, healthy: >2nd and <85th centiles, overweight: ⩾85th centile, obese: ⩾95th centile overweight-obese: ⩾85th centile.

b Only ethnic groups affected by the BMI adjustments presented.

**Table 4:** 10–11-year-old boys–summary of body mass index and weight categories using UK90 population thresholds^a^ by ethnic group before and after ethnic adjustments to BMI in the national child measurement programme (2012–13)

*Ethnic group*	N	*Median BMI, Kg m*^*−2*^*(LQ−UQ)*	*10–11-year-old boys prevalence, % (95 CI)*				*Overweight-obese*
			*Underweight*	*Healthy*	*Overweight*	*Obese*	
*Before BMI adjustment*							
White	160 278	17.8 (16.3–20.1)	0.9 (0.8–0.9)	66.3 (66.1–66.5)	14.1 (14.0–14.3)	18.7 (18.5–18.9)	32.8 (32.6–33.1)
Black	11 347	18.5 (16.7–21.4)	0.9 (0.7–1.1)	56.9 (56.0–57.8)	15.4 (14.8–16.1)	26.8 (26.0–27.6)	42.2 (41.3–43.1)
Black African	6172	18.6 (16.7–21.4)	1.0 (0.8–1.3)	55.8 (54.6–57.1)	15.9 (15.0–16.8)	27.2 (26.1–28.3)	43.1 (41.9–44.4)
Black Caribbean	2971	18.3 (16.7–21.3)	0.6 (0.3–0.9)	58.5 (56.7–60.2)	14.6 (13.3–15.9)	26.3 (24.7–27.9)	40.9 (39.2–42.7)
Black–Other	2204	18.5 (16.7–21.2)	1.0 (0.6–1.5)	57.6 (55.6–59.7)	15.1 (13.6–16.6)	26.2 (24.4–28.1)	41.3 (39.3–43.4)
South Asian	19 406	18.3 (16.0–21.4)	3.6 (3.4–3.9)	54.6 (53.9–55.3)	14.8 (14.3–15.3)	27.0 (26.4–27.6)	41.8 (41.1–42.5)
South Asian–Indian	5817	18.1 (15.9–21.1)	4.6 (4.0–5.1)	55.9 (54.6–57.2)	14.8 (13.9–15.7)	24.7 (23.6–25.8)	39.5 (38.3–40.8)
South Asian–Pakistani	9675	18.3 (16.0–21.4)	3.8 (3.5–4.2)	54.9 (53.9–55.9)	14.5 (13.8–15.2)	26.7 (25.9–27.6)	41.2 (40.2–42.2)
South Asian–Bangladeshi	3914	18.8 (16.3–22.0)	1.8 (0.3–0.6)	51.8 (50.2–53.4)	15.4 (14.2–16.5)	31.0 (29.6–32.5)	46.4 (44.8–48.0)
Other Asian	4325	18.4 (16.3–21.3)	2.4 (1.9–2.8)	55.2 (53.7–56.7)	15.8 (14.8–16.9)	26.6 (25.3–27.9)	42.4 (41.0–43.9)
Other ethnicity	12 914	18.2 (16.4–20.9)	1.0 (0.9–1.2)	60.1 (59.3–60.9)	15.2 (14.6–15.9)	23.6 (22.9–24.4)	38.9 (38.0–39.7)
Unknown	40 513	17.9 (16.3–20.4)	1.0 (0.9–1.1)	64.3 (63.8–64.8)	14.5 (14.2–14.9)	20.1 (19.7–20.5)	34.7 (34.2–35.1)
Overall	248 783	17.9 (16.3–20.4)	1.1 (1.1–1.2)	64.1 (63.9–64.3)	14.4 (14.3–14.5)	20.4 (20.2–20.5)	34.7 (34.6–34.9)
							
*After BMI adjustment* b							
White							
Black		17.6 (16.0–21.1)	2.1 (1.8–2.4)	66.0 (65.1–66.9)	13.4 (12.8–14.0)	18.5 (17.8–19.2)	31.9 (31.0–32.8)
Black African		17.7 (16.0–20.1)	2.2 (1.8–2.6)	65.6 (64.4–66.8)	13.5 (12.7–14.4)	18.7 (17.7–19.7)	32.2 (31.0–33.4)
Black Caribbean		17.4 (16.0–20.0)	1.7 (1.2–2.2)	66.3 (64.6–68.0)	13.0 (11.8–14.2)	18.9 (17.5–20.4)	31.9 (30.3–33.6)
Black–Other		17.5 (16.0–20.0)	2.4 (1.7–3.0)	66.7 (64.7–68.6)	13.7 (12.3–15.1)	17.3 (15.7–18.9)	31.0 (29.1–32.9)
South Asian		19.4 (17.1–22.6)	0.4 (0.3–0.5)	47.5 (46.8–48.2)	16.7 (16.2–17.3)	35.4 (34.7–36.1)	52.1 (51.4–52.9)
South Asian–Indian		19.2 (17.0–22.2)	0.4 (0.2–0.5)	49.5 (48.3–50.8)	16.8 (15.9–17.8)	33.3 (32.1–34.5)	50.1 (48.8–51.4)
South Asian–Pakistani		19.4 (17.1–22.5)	0.5 (0.3–0.6)	48.1 (47.1–49.1)	16.4 (15.7–17.2)	35.0 (34.1–36.0)	51.5 (50.5–52.5)
South Asian–Bangladeshi		19.9 (17.5–23.1)	0.2 (0.0–0.3)	42.9 (41.3–44.4)	17.4 (16.2–18.6)	39.5 (38.0–41.1)	56.9 (55.4–58.5)
Other Asian							
Other ethnicity							
Unknown							
Overall		17.9 (16.4–20.4)	0.9 (0.9–1.0)	64.0 (63.8–64.2)	14.5 (14.3–14.6)	20.6 (20.5–20.8)	35.1 (34.9–35.3)

a Population thresholds: underweight: ⩽2nd centile, healthy: >2nd and <85th centiles, overweight: ⩾85th centile, obese: ⩾95th centile Overweight-obese: ⩾85th centile.

b Only ethnic groups affected by the BMI adjustments presented.

**Table 3:** 4–5-year-old girls–summary of body mass index and weight categories using UK90 population thresholds^a^ by ethnic group before and after ethnic adjustments to BMI in the national child measurement programme (2012–13)

*Ethnic group*	N	*Median BMI, Kg m^−^*^*2*^*(LQ–UQ)*	*4–5-year-old girls prevalence, % (95% CI)*				*Overweight-obese*
			*Underweight*	*Healthy*	*Overweight*	*Obese*	
*Before BMI adjustment*							
White	188 663	16.0 (15.1–16.9)	0.4 (0.4–0.4)	78.7 (78.5–78.9)	12.8 (12.7–13.0)	8.1 (7.9–8.2)	20.9 (20.7–21.1)
Black	13 970	16.2 (15.2–17.4)	0.7 (0.5–0.8)	70.4 (69.7–71.2)	13.8 (13.2–14.4)	15.1 (14.5–15.7)	28.9 (28.2–29.7)
Black African	8520	16.2 (15.2–17.5)	0.7 (0.5–0.8)	68.8 (67.8–69.8)	14.6 (13.8–15.3)	16.0 (15.2–16.8)	30.6 (29.6–31.5)
Black Caribbean	2788	16.0 (15.0–17.3)	0.7 (0.4–1.0)	72.8 (71.2–74.5)	12.5 (11.3–13.7)	14.0 (12.7–15.3)	26.5 (24.8–28.1)
Black–Other	2662	16.1 (15.1–17.3)	0.6 (0.3–0.9)	73.1 (71.4–74.8)	12.7 (11.5–14.0)	13.6 (12.3–14.9)	26.3 (24.6–28.0)
South Asian	22 109	15.4 (14.5–16.7)	2.6 (2.4–2.8)	78.5 (78.0–79.1)	9.1 (8.7–9.5)	9.8 (9.4–10.2)	18.9 (18.4–19.4)
South Asian–Indian	7200	15.2 (14.3–16.4)	3.4 (3.0–3.8)	81.1 (80.2–82.0)	7.6 (7.0–8.2)	7.8 (7.2–8.5)	15.4 (14.6–16.3)
South Asian–Pakistani	10 855	15.5 (14.6–16.8)	2.3 (2.0–2.6)	77.7 (76.9–78.5)	9.8 (9.2–10.4)	10.2 (9.7–10.8)	20.0 (19.3–20.8)
South Asian–Bangladeshi	4054	15.6 (14.6–16.9)	1.9 (0.1–0.2)	76.1 (74.8–77.4)	10.1 (9.1–11.0)	11.9 (10.9–12.9)	22.0 (20.7–23.3)
Other Asian	6062	15.6 (14.7–16.8)	1.6 (1.3–2.0)	79.9 (78.9–80.9)	10.4 (9.6–11.2)	8.1 (7.4–8.8)	18.5 (17.5–19.5)
Other ethnicity	18 168	15.9 (15.0–17.0)	0.9 (0.7–1.0)	77.4 (76.8–78.0)	12.0 (11.5–12.5)	9.7 (9.3–10.1)	21.7 (21.1–22.3)
Unknown	36 040	15.9 (15.1–17.0)	0.6 (0.5–0.7)	78.1 (77.6–78.5)	12.6 (12.2–12.9)	8.8 (8.5–9.1)	21.4 (20.9–21.8)
Overall	285 012	15.9 (15.2–17.0)	0.7 (0.6–0.7)	78.2 (78.0–78.3)	12.5 (12.3–12.6)	8.7 (8.6–8.8)	21.2 (21.0–21.8)
							
After BMI adjustment^b^							
White							
Black		15.1 (14.3–16.1)	2.8 (2.6–3.1)	85.0 (84.4–85.6)	6.4 (6.0–6.8)	5.8 (5.4–6.1)	12.2 (11.6–12.7)
Black African		15.1 (14.3–16.2)	2.7 (2.4–3.0)	84.3 (83.6–85.1)	6.8 (6.3–7.4)	6.1 (5.6–6.6)	13.0 (12.2–13.7)
Black Caribbean		15.0 (14.2–16.0)	3.1 (2.5–3.8)	85.5 (84.2–86.8)	6.2 (5.3–7.1)	5.2 (4.4–6.0)	11.4 (10.2–12.5)
Black–Other		15.0 (14.2–16.0)	2.9 (2.3–3.6)	86.6 (85.3–87.9)	5.3 (4.5–6.2)	5.1 (4.3–6.0)	10.5 (9.3–11.6)
South Asian		16.5 (15.6–17.7)	0.2 (0.1–0.2)	65.0 (64.3–65.6)	17.2 (16.7–17.7)	17.7 (17.2–18.2)	34.9 (34.3–35.5)
South Asian–Indian		16.3 (15.4–17.5)	0.2 (0.1–0.3)	69.9 (68.9–71.0)	15.6 (14.7–16.4)	14.3 (13.5–15.1)	29.9 (28.8–30.9)
South Asian–Pakistani		16.6 (15.7–17.8)	0.2 (0.1–0.2)	62.7 (61.8–63.6)	18.3 (17.6–19.0)	18.8 (18.1–19.6)	37.2 (36.3–38.1)
South Asian–Bangladeshi		16.6 (15.7–18.0)	0.1 (0.0–0.2)	62.2 (60.7–63.7)	17.0 (15.8–18.2)	20.7 (19.4–21.9)	37.7 (36.2–39.2)
Other Asian							
Other ethnicity							
Unknown							
Overall		16.0 (15.1–17.0)	0.6 (0.5–0.6)	77.8 (77.7–78.0)	12.7 (12.6–12.8)	8.9 (8.8–9.0)	21.6 (21.5–21.8)

a Population thresholds: underweight: ⩽2nd centile, healthy: >2nd and <85th centiles, overweight: ⩾85th centile, obese: ⩾95th centile overweight-obese: ⩾85th centile.

b Only ethnic groups affected by the BMI adjustments presented.

**Table 2:** 4–5-year-old boys–summary of body mass index and weight categories using UK90 population thresholds^a^ by ethnic group before and after ethnic adjustments to BMI in the national child measurement programme (2012–13)

*Ethnic group*	N	*Median BMI, kg m*^*−2*^*(LQ–UQ)*	*4–5-year-old boys prevalence, % (95% CI)*				*Overweight-obese*
			*Underweight*	*Healthy*	*Overweight*	*Obese*	
*Before BMI adjustment*							
White	197 691	16.0 (15.3–16.9)	0.7 (0.6–0.7)	76.3 (76.1–76.5)	14.1 (13.9–14.2)	8.9 (8.8–9.1)	23.0 (22.8–23.2)
Black	14 468	16.2 (15.3–17.3)	1.1 (0.9–1.3)	68.1 (67.3–68.8)	14.9 (14.4–15.5)	15.9 (15.3–16.5)	30.8 (30.1–31.6)
Black African	9.003	16.3 (15.4–17.4)	1.1 (0.8–1.3)	65.8 (64.8–66.7)	15.8 (15.0–16.5)	17.4 (16.6–18.2)	33.2 (32.2–34.2)
Black Caribbean	2838	16.0 (15.2–17.0)	0.9 (0.6–1.3)	74.3 (72.7–76.0)	13.2 (12.0–14.5)	11.5 (10.3–12.7)	24.7 (23.1–26.3)
Black–other	2627	16.2 (15.3–17.2)	1.6 (1.1–2.0)	69.1 (67.4–70.9)	14.0 (12.7–15.3)	15.3 (13.9–16.7)	29.3 (27.6–31.1)
South Asian	23 191	15.5 (14.6–16.6)	4.3 (4.1–4.6)	76.3 (75.8–76.9)	8.3 (8.0–8.7)	11.0 (10.6–11.4)	19.3 (18.8–19.9)
South Asian–Indian	7474	15.3 (14.4–16.3)	6.1 (5.5–6.6)	77.6 (76.7–78.6)	7.1 (6.6–7.7)	9.2 (8.5–9.8)	16.3 (15.5–17.1)
South Asian–Pakistani	11 502	15.5 (14.7–16.6)	3.7 (3.3–4.0)	75.9 (75.1–76.7)	9.1 (8.5–9.6)	11.4 (10.8–12.0)	20.4 (19.7–21.2)
South Asian −Bangladeshi	4215	15.6 (14.7–16.7)	3.1 (0.1–0.2)	75.2 (73.9–76.5)	8.5 (7.7–9.4)	13.2 (12.2–14.2)	21.8 (20.5–23.0)
Other Asian	6184	15.7 (14.9–16.8)	2.6 (2.2–3.0)	75.9 (74.8–76.9)	10.7 (9.9–11.5)	10.8 (10.1–11.6)	21.5 (20.5–22.5)
Other ethnicity	18 832	15.9 (15.2–16.9)	1.2 (1.0–1.3)	75.4 (74.7–76.0)	12.9 (12.4–13.4)	10.6 (10.1–11.0)	23.5 (22.9–24.0)
Unknown	37 521	16.0 (15.2–16.9)	1.0 (0.9–1.1)	75.5 (75.1–76.0)	13.5 (13.1–13.8)	10.0 (9.7–10.3)	23.4 (23.0–23.9)
Overall	297 887	16.0 (15.2–16.9)	1.1 (1.1–1.1)	75.7 (75.6–75.9)	13.4 (13.3–13.6)	9.7 (9.6–9.8)	23.2 (23.0–23.3)
							
*After BMI adjustment*^b^							
White							
Black		15.1 (14.4–15.9)	4.3 (4.0–4.7)	85.1 (84.5–85.7)	5.8 (5.4–6.1)	4.8 (4.4–5.1)	10.5 (10.0–11.0)
Black African		15.2 (14.4–16.9)	4.1 (3.7–4.5)	84.1 (83.4–84.9)	6.4 (5.9–6.9)	5.4 (4.9–5.8)	11.8 (11.1–12.4)
Black Caribbean		15.0 (14.3–15.7)	4.2 (3.4–4.9)	88.5 (87.4–89.7)	4.4 (3.6–5.1)	2.9 (2.3–3.5)	7.3 (6.3–8.3)
Black–Other		15.1 (14.4–15.9)	5.3 (4.4–6.1)	84.8 (83.5–86.2)	5.0 (4.2–5.8)	4.9 (4.0–5.7)	9.9 (8.7–11.0)
South Asian		16.6 (15.7–17.7)	0.2 (0.2–0.3)	60.4 (59.8–61.1)	18.4 (17.9–18.9)	21.0 (20.5–21.5)	39.4 (38.7–40.0)
South Asian–Indian		16.4 (15.5–17.5)	0.3 (0.2–0.4)	65.2 (64.1–66.3)	16.7 (15.9–17.5)	17.8 (16.9–18.7)	34.5 (33.4–35.6)
South Asian–Pakistani		16.6 (15.8–17.8)	0.2 (0.1–0.2)	58.5 (57.6–59.4)	19.2 (18.5–19.9)	22.2 (21.4–23.0)	41.4 (40.5–42.3)
South Asian–Bangladeshi		16.7 (15.8–17.9)	0.2 (0.1–0.4)	57.4 (55.9–58.9)	19.1 (18.0–20.3)	23.3 (22.0–24.5)	42.4 (40.9–43.9)
Other Asian							
Other ethnicity							
Unknown							
Overall		16.0 (15.2–16.9)	0.9 (0.9–1.0)	75.3 (75.2–75.5)	13.8 (13.7–13.9)	10.0 (9.8–10.1)	23.7 (23.6–23.9)

a Population thresholds: underweight: ⩽2nd centile, healthy: >2nd and <85th centiles, overweight: ⩾85th centile, obese: ⩾95th centile overweight-obese: ⩾85th centile.

b Only ethnic groups affected by the BMI adjustments presented.
