# Supplementary material for: Patterns of childhood body mass index (BMI), overweight and obesity in South Asian and black participants in the English National child measurement programme: effect of applying BMI adjustments standardising for ethnic differences in BMI-body fatness associations
Source: Int J Obes (Lond). 2018 Feb 6;42(4):662–70. doi: 10.1038/ijo.2017.272 (PMC5815501; doi:10.1038/ijo.2017.272)
Supplement: Supplementary Material [file ijo2017272x1.docx]

SUPPLEMENTARY FIGURE 1: TOP 20 RANKED LOCAL AUTHORITIES OF ENGLAND BASED ON PREVALENCE OF OVERWEIGHT-OBESITY IN 4-5 YEAR OLD BOYS IN THE NATIONAL CHILD MEASUREMENT PROGRAMME (2012-13) BEFORE (LEFT) AND AFTER (RIGHT) BMI ADJUSTMENTS

AFTER ADJUSTMENT:

1 - TOWER HAMLETS

2 - WOLVERHAMPTON

3 - BRADFORD

4 - NEWCASTLE UPON TYNE

5 - OLDHAM

6 - LIVERPOOL

7 - LUTON

8 - PETERBOROUGH

9 - REDBRIDGE

10 - NORTH TYNESIDE

11 - BLACKBURN WITH DARWEN

12 - HOUNSLOW

13 - BIRMINGHAM

14 - WALSALL

15 - DEVON

16 - MANCHESTER

17 - NEWHAM

18 - BLACKPOOL

19 - ROCHDALE

20 - KIRKLEES

21 - GLOUCESTERSHIRE

22 - LINCOLNSHIRE

27 - PLYMOUTH

28 - HARTLEPOOL

42 - CITY OF WESTMINSTER

52 - BEXLEY

53 - HACKNEY

60 - ENFIELD

81 - BARKING & DAGENHAM

100 - HAMMERSMITH & FULHAM

124 - GREENWICH

130 - SOUTHWARK

137 - LEWISHAM

BEFORE ADJUSTMENT:

1 - LIVERPOOL

2 - WOLVERHAMPTON

3 - NEWCASTLE UPON TYNE

4 - NORTH TYNESIDE

5 - HACKNEY

6 - SOUTHWARK

7 - GREENWICH

8 - BARKING & DAGENHAM

9 - DEVON COUNTY

10 - ENFIELD

11 - MANCHESTER

12 - LEWISHAM

13 - BLACKPOOL

14 - BEXLEY

15 - HAMMERSMITH & FULHAM

16 - GLOUCESTERSHIRE

17 - LINCOLNSHIRE

18 - PLYMOUTH

19 - HARTLEPOOL

20 - CITY OF WESTMINSTER

27 - NEWHAM

29 - PETERBOROUGH

31 - HOUNSLOW

38 - OLDHAM

43 - WALSALL

49 - TOWER HAMLETS

60 - LUTON

75 - BIRMINGHAM

87 - BRADFORD

96 - BLACKBURN WITH DARWEN

99 - KIRKLEES

101 - ROCHDALE

111 - REDBRIDGE

Line colours represent the prevalence of specific ethnic minority groups in individual boroughs.

Grey = South Asian & Blacks < 20%, Green = South Asian ≥ 20% & Blacks < 20%, Red = Blacks ≥ 20% & South Asian < 20%, Blue = South Asian & Blacks ≥ 20%

SUPPLEMENTARY FIGURE 2: TOP 20 RANKED LOCAL AUTHORITIES OF ENGLAND BASED ON PREVALENCE OF OVERWEIGHT-OBESITY IN 4-5 YEAR OLD GIRLS IN THE NATIONAL CHILD MEASUREMENT PROGRAMME (2012-13) BEFORE (LEFT) AND AFTER (RIGHT) BMI ADJUSTMENTS

BEFORE ADJUSTMENT:

1 - LIVERPOOL

2 - BEXLEY

3 - KNOWSLEY

4 - HALTON

5 - SOUTH TYNESIDE

6 - GREENWICH

7 - SOUTHWARK

8 - ENFIELD

9 - BLACKPOOL

10 - WOLVERHAMPTON

11 - LINCOLNSHIRE

12 - NORTH LINCOLNSHIRE

13 - HACKNEY

14 - ST HELENS

15 - HARTLEPOOL

16 - STOCKTON-ON-TEES

17 - NEWHAM

18 - MANCHESTER

19 - PORTSMOUTH

20 - NORTH TYNESIDE

21 - STOKE-ON-TRENT

23 - DERBY

24 - NEWCASTLE UPON TYNE

34 - BIRMINGHAM

37 - SLOUGH

38 - TOWER HAMLETS

39 - OLDHAM

43 - LEICESTER

89 - BLACKBURN WITH DARWEN

91 - LUTON

99 - BRADFORD

AFTER ADJUSTMENT:

1 - TOWER HAMLETS

2 - OLDHAM

3 - BLACKBURN WITH DARWEN

4 - BIRMINGHAM

5 - NEWHAM

6 - LIVERPOOL

7 - BRADFORD

8 - KNOWSLEY

9 - SLOUGH

10 - LEICESTER

11 - HALTON

12 - DERBY

13 - WOLVERHAMPTON

14 - LUTON

15 - SOUTH TYNESIDE

16 - STOKE-ON-TRENT

17 - BLACKPOOL

18 - NEWCASTLE UPON TYNE

19 - NORTH LINCOLNSHIRE

20 - BEXLEY

21 - LINCOLNSHIRE

22 - ST HELENS

24 - NORTH TYNESIDE

25 - STOCKTON-ON-TEES

27 - MANCHESTER

28 - HARTLEPOOL

32 - PORTSMOUTH

70 - ENFIELD

82 - HACKNEY

94 - GREENWICH

124 - SOUTHWARK

Line colours represent the prevalence of specific ethnic minority groups in individual boroughs.

Grey = South Asian & Blacks < 20%, Green = South Asian ≥ 20% & Blacks < 20%, Red = Blacks ≥ 20% & South Asian < 20%, Blue = South Asian & Blacks ≥ 20%

SUPPLEMENTARY FIGURE 3: TOP 20 RANKED LOCAL AUTHORITIES OF ENGLAND BASED ON PREVALENCE OF OVERWEIGHT-OBESITY IN 10-11 YEAR OLD BOYS IN THE NATIONAL CHILD MEASUREMENT PROGRAMME (2012-13) BEFORE (LEFT) AND AFTER (RIGHT) BMI ADJUSTMENTS

AFTER ADJUSTMENT:

1 - TOWER HAMLETS

2 - NEWHAM

3 - CITY OF WESTMINSTER

4 - HOUNSLOW

5 - REDBRIDGE

6 - CITY OF WOLVERHAMPTON

7 - BIRMINGHAM

8 - SANDWELL

9 - LUTON

10 - SLOUGH

11 - NEWCASTLE UPON TYNE

12 - BRADFORD

13 - WALTHAM FOREST

14 - BEXLEY

15 - SOUTH TYNESIDE

16 - LIVERPOOL

17 - WALSALL

18 - SOUTHWARK

19 - EALING

20 - KNOWSLEY

23 - BARKING & DAGENHAM

24 - BRENT

25 - HACKNEY

28 - HARINGEY

31 - ENFIELD

47 - LAMBETH

BEFORE ADJUSTMENT:

1 - CITY OF WESTMINSTER

2 - TOWER HAMLETS

3 - SOUTHWARK

4 - NEWHAM

5 - HOUNSLOW

6 - CITY OF WOLVERHAMPTON

7 - LAMBETH

8 - HARINGEY

9 - HACKNEY

10 - WALTHAM FOREST

11 - BEXLEY

12 - EALING

13 - LIVERPOOL

14 - BRENT

15 - SANDWELL

16 - ENFIELD

17 - BARKING & DAGENHAM

18 - KNOWSLEY

19 - SOUTH TYNESIDE

20 - NEWCASTLE UPON TYNE

21 - BIRMINGHAM

22 - WALSALL

23 - REDBRIDGE

30 - LUTON

40 - BRADFORD

45 - SLOUGH

Line colours represent the prevalence of specific ethnic minority groups in individual boroughs.

Grey = South Asian & Blacks < 20%, Green = South Asian ≥ 20% & Blacks < 20%, Red = Blacks ≥ 20% & South Asian < 20%, Blue = South Asian & Blacks ≥ 20%

SUPPLEMENTARY FIGURE 4: TOP 20 RANKED LOCAL AUTHORITIES OF ENGLAND BASED ON PREVALENCE OF OVERWEIGHT-OBESITY IN 10-11 YEAR OLD GIRLS IN THE NATIONAL CHILD MEASUREMENT PROGRAMME (2012-13) BEFORE (LEFT) AND AFTER (RIGHT) BMI ADJUSTMENTS

AFTER ADJUSTMENT:

1 - SOUTHWARK

2 - NEWHAM

3 - TOWER HAMLETS

4 - SANDWELL

5 - STOKE-ON-TRENT

6 - MANCHESTER

7 - CITY OF WOLVERHAMPTON

8 - BIRMINGHAM

9 - HALTON

10 - HACKNEY

11 - LUTON

12 - WALSALL

13 - BEXLEY

14 - DUDLEY

15 - BARKING & DAGENHAM

16 - HOUNSLOW

17 - HARTLEPOOL

18 - BRENT

19 - SUNDERLAND

20 - LIVERPOOL

24 - CROYDON

26 - ENFIELD

29 - HARINGEY

30 - GREENWICH

50 - LEWISHAM

62 - LAMBETH

BEFORE ADJUSTMENT:

1 - SOUTHWARK

2 - HACKNEY

3 - SANDWELL

4 - NEWHAM

5 - MANCHESTER

6 - BARKING & DAGENHAM

7 - HALTON

8 - STOKE-ON-TRENT

9 - GREENWICH

10 - BRENT

11 - CITY OF WOLVERHAMPTON

12 - CROYDON

13 - BEXLEY

14 - LEWISHAM

15 - ENFIELD

16 - HARINGEY

17 - LAMBETH

18 - LIVERPOOL

19 - HARTLEPOOL

20 - TOWER HAMLETS

22 - DUDLEY

23 - SUNDERLAND

24 - WALSALL

25 - BIRMINGHAM

28 - HOUNSLOW

29 - LUTON

Line colours represent the prevalence of specific ethnic minority groups in individual boroughs.

Grey = South Asian & Blacks < 20%, Green = South Asian ≥ 20% & Blacks < 20%, Red = Blacks ≥ 20% & South Asian < 20%, Blue = South Asian & Blacks ≥ 20%

SUPPLEMENTARY TABLE 1: BODY MASS INDEX ADJUSTMENT FACTORS FOR BLACK AFRICAN CHILDREN BY SEX AND AGE GROUP

|  | **Boys** | |  | **Girls** | |
| --- | --- | --- | --- | --- | --- |
|  | **Adjusted BMI** | |  | **Adjusted BMI** | |
|  | **(kg/m^2^)** | |  | **(kg/m^2^)** | |
| **BMI** | **4-5 years** | **10-11 years** |  | **4-5 years** | **10-11 years** |
| **(kg/m^2^)** |  |  |  |  |  |
| **13** | 12.57 | 12.76 |  | 12.51 | 12.78 |
| **14** | 13.37 | 13.64 |  | 13.31 | 13.67 |
| **15** | 14.17 | 14.52 |  | 14.12 | 14.55 |
| **16** | 14.97 | 15.40 |  | 14.92 | 15.44 |
| **17** | 15.77 | 16.29 |  | 15.72 | 16.32 |
| **18** | 16.57 | 17.17 |  | 16.52 | 17.21 |
| **19** | 17.37 | 18.05 |  | 17.33 | 18.10 |
| **20** | 18.17 | 18.93 |  | 18.13 | 18.98 |
| **21** | 18.97 | 19.81 |  | 18.93 | 19.87 |
| **22** | 19.77 | 20.69 |  | 19.74 | 20.76 |
| **23** | 20.57 | 21.57 |  | 20.54 | 21.64 |
| **24** | 21.16 | 22.45 |  | 21.34 | 22.53 |
| **25** | 22.16 | 23.33 |  | 22.14 | 23.42 |

SUPPLEMENTARY TABLE 2: LOCAL AUTHORITY PREVALENCES BEFORE AND AFTER BMI ADJUSTMENT BY SEX AND AGE GROUP IN THE NATIONAL CHILD MEASUREMENT PROGRAMME (2012-13)

|  | **Area** | **Boys** | | | | **Girls** | | | |
| --- | --- | --- | --- | --- | --- | --- | --- | --- | --- |
|  |  | **Overweight-Obese** | | | | **Overweight-Obese** | | | |
|  |  | **4-5 Years old** | | **10-11 Years old** | | **4-5 Years old** | | **10-11 Years old** | |
|  |  | **Before** | **After** | **Before** | **After** | **Before** | **After** | **Before** | **After** |
|  | BARKING AND DAGENHAM (LONDON BORO) | 27·5% | 23·3% | 40·9% | 40·2% | 23·9% | 20·9% | 38·7% | 37·4% |
|  | BARNET (LONDON BORO) | 25·4% | 23·9% | 37·3% | 36·5% | 21·7% | 20·9% | 29·4% | 29·2% |
|  | BARNSLEY DISTRICT | 22·9% | 22·8% | 36·3% | 36·1% | 23·6% | 23·6% | 34·4% | 34·4% |
|  | BATH AND NORTH EAST SOMERSET | 24·2% | 24·2% | 27·6% | 27·6% | 22·7% | 22·9% | 25·0% | 25·0% |
|  | BEDFORD | 24·2% | 26·2% | 36·4% | 37·5% | 23·5% | 24·6% | 32·7% | 33·0% |
|  | BEXLEY (LONDON BORO) | 26·7% | 24·4% | 41·5% | 41·2% | 26·9% | 25·0% | 38·1% | 37·7% |
|  | BIRMINGHAM DISTRICT | 23·1% | 28·2% | 40·7% | 43·6% | 23·4% | 27·3% | 36·3% | 38·9% |
|  | BLACKBURN WITH DARWEN | 22·5% | 28·4% | 32·9% | 37·6% | 20·9% | 27·6% | 30·4% | 34·9% |
|  | BLACKPOOL | 26·9% | 27·1% | 37·3% | 37·3% | 25·2% | 25·4% | 33·0% | 33·0% |
|  | BOLTON DISTRICT | 20·9% | 24·3% | 36·8% | 38·8% | 19·2% | 21·5% | 34·3% | 36·0% |
|  | BOURNEMOUTH | 22·8% | 23·4% | 32·3% | 32·5% | 20·4% | 20·8% | 30·8% | 31·0% |
|  | BRACKNELL FOREST | 18·6% | 18·3% | 30·1% | 29·7% | 20·2% | 20·0% | 23·6% | 23·8% |
|  | BRADFORD DISTRICT | 22·8% | 30·8% | 37·5% | 41·3% | 20·4% | 27·0% | 33·1% | 36·4% |
|  | BRENT (LONDON BORO) | 22·4% | 22·7% | 41·2% | 40·1% | 22·3% | 21·3% | 38·4% | 37·1% |
|  | BROMLEY (LONDON BORO) | 22·6% | 20·5% | 33·6% | 32·8% | 19·7% | 18·3% | 30·3% | 29·4% |
|  | BUCKINGHAMSHIRE COUNTY | 19·6% | 21·3% | 30·0% | 30·6% | 18·5% | 20·0% | 26·5% | 27·1% |
|  | BURY DISTRICT | 21·6% | 24·3% | 34·8% | 35·7% | 17·0% | 18·7% | 31·9% | 33·3% |
|  | CALDERDALE DISTRICT | 21·5% | 25·0% | 33·1% | 34·7% | 21·2% | 24·2% | 28·3% | 29·2% |
|  | CAMBRIDGESHIRE COUNTY | 21·4% | 22·1% | 29·5% | 29·7% | 19·0% | 19·5% | 28·5% | 28·6% |
|  | CAMDEN (LONDON BORO) | 22·3% | 23·5% | 36·9% | 38·2% | 19·5% | 18·7% | 34·2% | 33·9% |
|  | CENTRAL BEDFORDSHIRE | 20·2% | 20·0% | 29·2% | 28·9% | 19·2% | 19·0% | 27·7% | 27·7% |
|  | CHESHIRE EAST | 21·7% | 21·9% | 30·7% | 30·8% | 19·6% | 19·7% | 28·2% | 28·3% |
|  | CHESHIRE WEST AND CHESTER | 24·2% | 24·4% | 31·8% | 31·8% | 23·8% | 24·1% | 31·3% | 31·3% |
|  | CITY AND COUNTY OF THE CITY OF LONDON** | 0% | 0% | 20·0% | 20·0% | 0% | 0% | 0% | 0% |
|  | CITY OF WESTMINSTER (LONDON BORO) | 25·8% | 25% | 46·3% | 45·5% | 21·0% | 20·7% | 31·7% | 31·0% |
|  | CITY OF BRISTOL | 22·0% | 21·5% | 36·1% | 36·1% | 21·9% | 21·7% | 31·9% | 31·7% |
|  | CITY OF DERBY | 23·3% | 25·2% | 33·0% | 34·6% | 23·9% | 25·8% | 33·5% | 35·1% |
|  | CITY OF KINGSTON UPON HULL | 25·3% | 25·3% | 37·0% | 37·3% | 20·6% | 20·9% | 35·5% | 35·4% |
|  | CITY OF LEICESTER | 21·3% | 24·9% | 36·9% | 40·6% | 22·8% | 26·5% | 32·6% | 34·6% |
|  | CITY OF NOTTINGHAM | 23·1% | 22·8% | 35·6% | 36·5% | 22·9% | 22·7% | 34·6% | 35·0% |
|  | CITY OF PETERBOROUGH | 25·3% | 28·5% | 35·7% | 35·8% | 21·5% | 23·6% | 32·5% | 32·8% |
|  | CITY OF PLYMOUTH | 26·2% | 26·1% | 34·3% | 34·3% | 23·4% | 23·3% | 29·7% | 29·7% |
|  | CITY OF PORTSMOUTH | 24·2% | 24·2% | 38·2% | 38·2% | 24·2% | 24·2% | 32·8% | 32·8% |
|  | CITY OF SOUTHAMPTON | 23·5% | 24·5% | 33·9% | 34·1% | 21·0% | 21·7% | 35·1% | 35·2% |
|  | CITY OF STOKE-ON-TRENT | 24·4% | 26·3% | 37·6% | 38·8% | 24·0% | 25·4% | 38·6% | 39·9% |
|  | CITY OF WOLVERHAMPTON DISTRICT | 28·9% | 30·8% | 42·9% | 43·8% | 25·1% | 25·6% | 38·3% | 39·0% |
|  | CORNWALL | 25·7% | 25·7% | 32·5% | 32·5% | 22·5% | 22·5% | 29·2% | 29·2% |
|  | COUNTY DURHAM | 22·6% | 22·6% | 37·2% | 37·3% | 21·1% | 21·2% | 34·7% | 34·7% |
|  | COUNTY OF HEREFORDSHIRE | 21·2% | 21·2% | 30·7% | 30·7% | 20·3% | 20·3% | 27·6% | 27·6% |
|  | COVENTRY DISTRICT | 21·5% | 21·9% | 34·5% | 35·7% | 19·7% | 20·4% | 34·3% | 34·7% |
|  | CROYDON (LONDON BORO) | 25·4% | 20·8% | 38·3% | 36·0% | 22·2% | 18·6% | 38·1% | 36·3% |
|  | CUMBRIA COUNTY | 24·0% | 24·0% | 38·6% | 38·6% | 21·5% | 21·5% | 34·8% | 34·8% |
|  | DARLINGTON | 24·4% | 24·4% | 33·5% | 33·5% | 19·8% | 20·0% | 33·5% | 33·7% |
|  | DERBYSHIRE COUNTY | 22·6% | 22·7% | 33·1% | 33·2% | 19·7% | 19·9% | 30·9% | 30·9% |
|  | DEVON COUNTY | 27·4% | 27·6% | 31·0% | 31·0% | 22·0% | 22·1% | 28·4% | 28·4% |
|  | DONCASTER DISTRICT | 22·5% | 22·7% | 36·2% | 36·4% | 21·6% | 21·6% | 30·6% | 30·8% |
|  | DORSET COUNTY | 21·4% | 21·4% | 32·1% | 32·1% | 18·4% | 18·4% | 25·8% | 25·8% |
|  | DUDLEY DISTRICT | 24·6% | 26·1% | 37·9% | 38·6% | 22·5% | 23·3% | 36·4% | 37·5% |
|  | EALING (LONDON BORO) | 22·8% | 25·4% | 41·4% | 40·9% | 22·1% | 21·6% | 34·6% | 34·4% |
|  | EAST RIDING OF YORKSHIRE | 20·7% | 20·8% | 34·5% | 34·5% | 21·2% | 21·2% | 30·7% | 30·8% |
|  | EAST SUSSEX COUNTY | 21·9% | 21·9% | 32·3% | 32·4% | 19·7% | 19·7% | 27·4% | 27·7% |
|  | ENFIELD (LONDON BORO) | 27·2% | 24·2% | 40·9% | 38·7% | 25·2% | 21·6% | 37·3% | 36·0% |
|  | ESSEX COUNTY | 21·5% | 21·3% | 32·4% | 32·3% | 19·2% | 19·2% | 29·1% | 29·1% |
|  | GATESHEAD DISTRICT | 22·9% | 22·8% | 38·1% | 38·1% | 20·7% | 20·7% | 33·6% | 33·7% |
|  | GLOUCESTERSHIRE COUNTY | 26·6% | 26·6% | 33·7% | 33·9% | 22·6% | 22·5% | 32·0% | 32·0% |
|  | GREENWICH (LONDON BORO) | 28·0% | 21·1% | 40·0% | 37·3% | 25·3% | 20·6% | 38·4% | 35·5% |
|  | HACKNEY (LONDON BORO) | 28·2% | 24·4% | 41·7% | 39·9% | 24·8% | 21·1% | 41·2% | 38·4% |
|  | HALTON | 23·8% | 23·8% | 34·6% | 34·6% | 26·0% | 26·0% | 38·7% | 38·7% |
|  | HAMMERSMITH AND FULHAM (LONDON BORO) | 26·7% | 22·3% | 39·3% | 38·1% | 23·7% | 19·3% | 33·3% | 30·9% |
|  | HAMPSHIRE COUNTY | 20·9% | 20·9% | 29·7% | 29·7% | 19·4% | 19·4% | 25·9% | 25·9% |
|  | HARINGEY (LONDON BORO) | 24·2% | 19·6% | 41·7% | 39·5% | 21·1% | 18·6% | 37·1% | 35·5% |
|  | HARROW (LONDON BORO) | 22·2% | 25·0% | 38·7% | 39·8% | 20·2% | 23·1% | 29·3% | 29·6% |
|  | HARTLEPOOL | 26·0% | 26·0% | 39·7% | 39·7% | 24·4% | 24·4% | 36·8% | 37·2% |
|  | HAVERING (LONDON BORO) | 20·1% | 19·1% | 37·5% | 36·9% | 21·7% | 20·7% | 32·6% | 31·9% |
|  | HERTFORDSHIRE COUNTY | 20·8% | 21·2% | 30·6% | 30·8% | 18·7% | 19·0% | 26·8% | 26·9% |
|  | HILLINGDON (LONDON BORO) | 22·8% | 23·7% | 35·0% | 36·2% | 20·0% | 21·7% | 34·3% | 34·7% |
|  | HOUNSLOW (LONDON BORO) | 25·0% | 28·3% | 43·0% | 45·2% | 21·1% | 23·1% | 35·8% | 37·3% |
|  | ISLES OF SCILLY ** | 33·3% | 33·3% | 14·3% | 14·3% | 20·0% | 20·0% | 0% | 0% |
|  | ISLE OF WIGHT | 23·9% | 23·9% | 35·8% | 35·8% | 24·0% | 24·0% | 29·6% | 29·6% |
|  | ISLINGTON (LONDON BORO) | 24·1% | 21·0% | 37·7% | 36·1% | 22·3% | 20·4% | 35·3% | 33·0% |
|  | KENSINGTON AND CHELSEA (LONDON BORO) | 18·8% | 17·0% | 35·8% | 34·2% | 21·4% | 19·2% | 30·6% | 28·2% |
|  | KENT COUNTY | 22·6% | 22·7% | 34·0% | 33·9% | 20·9% | 21·0% | 31·3% | 31·4% |
|  | KINGSTON UPON THAMES (LONDON BORO) | 16·4% | 17·2% | 33·2% | 33·8% | 15·7% | 16·5% | 27·1% | 27·4% |
|  | KIRKLEES DISTRICT | 22·4% | 27·1% | 33·3% | 34·9% | 20·7% | 24·3% | 30·8% | 32·8% |
|  | KNOWSLEY DISTRICT | 25·6% | 25·6% | 40·9% | 40·9% | 26·7% | 26·7% | 36·5% | 36·5% |
|  | LAMBETH (LONDON BORO) | 23·6% | 16·6% | 41·8% | 37·1% | 23·4% | 18·4% | 37·1% | 33·5% |
|  | LANCASHIRE COUNTY | 24·6% | 26·3% | 34·2% | 34·8% | 22·4% | 23·6% | 30·6% | 31·1% |
|  | LEEDS DISTRICT | 23·3% | 23·3% | 34·7% | 35·6% | 22·4% | 23·0% | 32·9% | 33·5% |
|  | LEICESTERSHIRE COUNTY | 22·4% | 23·3% | 32·8% | 33·2% | 19·2% | 19·9% | 29·7% | 30·2% |
|  | LEWISHAM (LONDON BORO) | 27·0% | 19·1% | 39·1% | 35·8% | 22·9% | 17·2% | 37·5% | 34·3% |
|  | LINCOLNSHIRE COUNTY | 26·4% | 26·4% | 34·5% | 34·5% | 24·9% | 24·9% | 35·2% | 35·2% |
|  | LIVERPOOL DISTRICT | 30·1% | 30·0% | 41·3% | 41·1% | 27·1% | 27·1% | 36·9% | 36·6% |
|  | LUTON | 23·8% | 29·4% | 38·8% | 42·1% | 20·8% | 25·6% | 35·5% | 38·0% |
|  | MANCHESTER DISTRICT | 27·1% | 27·6% | 4·00% | 40·4% | 24·3% | 24·4% | 38·9% | 39·3% |
|  | MEDWAY | 24·0% | 24·2% | 34·1% | 34·8% | 22·4% | 22·3% | 31·6% | 31·7% |
|  | MERTON (LONDON BORO) | 22·1% | 22·0% | 36·4% | 36·4% | 20·0% | 18·5% | 33·5% | 32·6% |
|  | MIDDLESBROUGH * | 27·3% | 29·7% | 35·2% | 36·0% | 25·8% | 27·5% | 37·4% | 38·8% |
|  | MILTON KEYNES | 23·6% | 22·4% | 34·0% | 34·1% | 20·8% | 21·2% | 30·7% | 29·6% |
|  | NEWCASTLE UPON TYNE DISTRICT | 28·6% | 30·1% | 40·8% | 41·3% | 23·9% | 25·2% | 34·7% | 35·4% |
|  | NEWHAM (LONDON BORO) | 25·3% | 27·6% | 43·9% | 45·8% | 24·3% | 27·1% | 39·9% | 41·2% |
|  | NORFOLK COUNTY | 24·7% | 24·6% | 33·8% | 33·7% | 21·2% | 21·2% | 30·4% | 30·4% |
|  | NORTHAMPTONSHIRE COUNTY | 22·8% | 22·7% | 33·9% | 33·9% | 19·7% | 19·6% | 31·5% | 31·5% |
|  | NORTHUMBERLAND | 23·4% | 23·4% | 33·3% | 33·3% | 21·8% | 21·9% | 28·7% | 28·7% |
|  | NORTH EAST LINCOLNSHIRE | 24·3% | 24·3% | 34·5% | 34·5% | 21·8% | 21·8% | 33·5% | 33·5% |
|  | NORTH LINCOLNSHIRE | 24·8% | 25·4% | 36·1% | 36·5% | 24·8% | 25·1% | 30·6% | 31·1% |
|  | NORTH SOMERSET | 25·7% | 25·8% | 32·2% | 32·1% | 22·9% | 23·0% | 30·0% | 30·0% |
|  | NORTH TYNESIDE DISTRICT | 28·5% | 28·5% | 36·0% | 36·0% | 24·2% | 24·5% | 34·2% | 34·3% |
|  | NORTH YORKSHIRE COUNTY | 22·8% | 22·9% | 29·1% | 29·2% | 19·6% | 19·7% | 27·7% | 27·7% |
|  | NOTTINGHAMSHIRE COUNTY | 22·3% | 22·8% | 33·1% | 33·2% | 20·6% | 20·7% | 29·1% | 29·3% |
|  | OLDHAM DISTRICT | 24·6% | 30·0% | 36·2% | 37·8% | 23·0% | 28·0% | 31·8% | 33·0% |
|  | OXFORDSHIRE COUNTY | 19·8% | 19·9% | 30·1% | 30·2% | 16·8% | 17·0% | 26·9% | 27·0% |
|  | POOLE | 23·7% | 23·9% | 28·8% | 28·8% | 22·6% | 22·8% | 26·7% | 26·7% |
|  | READING | 23·1% | 23·1% | 33·3% | 34·2% | 20·9% | 20·9% | 35·9% | 36·2% |
|  | REDBRIDGE (LONDON BORO) | 21·9% | 28·5% | 40·1% | 43·9% | 19·5% | 24·4% | 32·1% | 35·0% |
|  | REDCAR AND CLEVELAND * | 33·2% | 33·2% | 42·9% | 42·9% | 31·4% | 31·4% | 37·4% | 37·4% |
|  | RICHMOND UPON THAMES (LONDON BORO) | 18·2% | 19·0% | 30·1% | 29·9% | 14·3% | 14·5% | 22·1% | 22·6% |
|  | ROCHDALE DISTRICT | 22·3% | 27·1% | 33·5% | 35·1% | 21·3% | 23·9% | 33·9% | 36·4% |
|  | ROTHERHAM DISTRICT | 23·4% | 24·8% | 36·7% | 37·5% | 20·9% | 22·0% | 33·8% | 34·5% |
|  | RUTLAND ** | 26·0% | 26·0% | 25·6% | 25·6% | 19·9% | 19·9% | 22·1% | 22·1% |
|  | SALFORD DISTRICT | 23·6% | 23·2% | 35·9% | 35·5% | 22·4% | 22·1% | 36·0% | 35·9% |
|  | SANDWELL DISTRICT | 22·1% | 25·5% | 41·0% | 42·2% | 21·5% | 23·8% | 40·1% | 40·5% |
|  | SEFTON DISTRICT | 25·3% | 25·3% | 35·3% | 35·3% | 21·5% | 21·5% | 35·1% | 35·1% |
|  | SHEFFIELD DISTRICT | 20·7% | 21·9% | 35·3% | 35·8% | 18·4% | 19·1% | 32·1% | 32·5% |
|  | SHROPSHIRE | 23·8% | 23·8% | 34·2% | 34·2% | 20·0% | 20·1% | 30·8% | 30·8% |
|  | SLOUGH | 20·9% | 22·7% | 37·0% | 41·7% | 23·2% | 26·5% | 31·3% | 33·8% |
|  | SOLIHULL DISTRICT | 21·3% | 23·1% | 29·5% | 30·6% | 16·8% | 17·4% | 27·1% | 28·3% |
|  | SOMERSET COUNTY | 24·7% | 24·7% | 29·7% | 29·7% | 21·8% | 22·0% | 28·7% | 28·7% |
|  | SOUTHEND-ON-SEA | 24·3% | 24·0% | 33·6% | 33·9% | 21·3% | 21·5% | 30·0% | 30·1% |
|  | SOUTHWARK (LONDON BORO) | 28·1% | 20·3% | 44·4% | 40·9% | 25·3% | 18·8% | 44·1% | 42·2% |
|  | SOUTH GLOUCESTERSHIRE | 17·7% | 17·9% | 30·6% | 30·5% | 15·8% | 15·9% | 29·8% | 30·0% |
|  | SOUTH TYNESIDE DISTRICT | 22·8% | 23·3% | 40·8% | 41·1% | 25·4% | 25·4% | 34·1% | 34·8% |
|  | STAFFORDSHIRE COUNTY | 24·1% | 24·5% | 35·0% | 35·3% | 23·6% | 23·7% | 32·0% | 32·2% |
|  | STOCKPORT DISTRICT | 23·8% | 24·6% | 30·9% | 31·1% | 19·5% | 19·9% | 31·4% | 32·1% |
|  | STOCKTON-ON-TEES | 21·2% | 21·2% | 35·7% | 35·7% | 24·4% | 24·5% | 34·1% | 34·1% |
|  | ST HELENS DISTRICT | 24·5% | 24·5% | 36·9% | 36·9% | 24·6% | 24·6% | 35·0% | 35·0% |
|  | SUFFOLK COUNTY | 22·0% | 22·3% | 31·9% | 31·9% | 19·9% | 19·9% | 28·9% | 29·0% |
|  | SUNDERLAND DISTRICT | 25·4% | 25·7% | 34·7% | 34·9% | 22·7% | 23·2% | 36·4% | 36·8% |
|  | SURREY COUNTY | 18·7% | 19·1% | 27·9% | 28·2% | 16·6% | 16·8% | 25·1% | 25·4% |
|  | SUTTON (LONDON BORO) | 21·4% | 21·3% | 35·3% | 36·1% | 18·4% | 18·7% | 30·3% | 30·5% |
|  | SWINDON | 23·3% | 23·8% | 34·7% | 34·9% | 23·3% | 23·7% | 30·8% | 31·9% |
|  | TAMESIDE DISTRICT | 23·0% | 23·0% | 34·3% | 34·3% | 23·6% | 23·6% | 32·2% | 32·5% |
|  | TELFORD AND WREKIN | 24·6% | 25·3% | 36·5% | 36·9% | 23·7% | 24·5% | 33·3% | 33·7% |
|  | THE CITY OF BRIGHTON AND HOVE | 22·7% | 22·2% | 27·8% | 27·5% | 19·8% | 19·8% | 25·5% | 25·3% |
|  | THURROCK | 23·8% | 21·3% | 37·1% | 35·6% | 20·3% | 18·8% | 33·6% | 32·5% |
|  | TORBAY * | 29·2% | 29·2% | 39·4% | 39·4% | 26·9% | 26·9% | 37·8% | 37·8% |
|  | TOWER HAMLETS (LONDON BORO) | 24·2% | 35·5% | 45·9% | 51·7% | 23·2% | 32·0% | 36·7% | 41·1% |
|  | TRAFFORD DISTRICT | 18·9% | 18·9% | 34·1% | 34·1% | 19·2% | 19·1% | 31·8% | 31·9% |
|  | WAKEFIELD DISTRICT | 24·1% | 24·4% | 34·6% | 34·6% | 21·1% | 21·7% | 33·3% | 33·8% |
|  | WALSALL DISTRICT | 24·3% | 28·2% | 40·4% | 41·0% | 21·3% | 23·7% | 36·3% | 37·8% |
|  | WALTHAM FOREST (LONDON BORO) | 22·7% | 23·4% | 41·6% | 41·2% | 19·4% | 19·7% | 34·3% | 33·5% |
|  | WANDSWORTH (LONDON BORO) | 23·3% | 21·8% | 39·8% | 37·6% | 20·8% | 18·6% | 30·7% | 29·6% |
|  | WARRINGTON | 23·0% | 23·7% | 33·2% | 33·4% | 20·7% | 20·9% | 29·9% | 30·1% |
|  | WARWICKSHIRE COUNTY | 21·0% | 21·7% | 31·8% | 32·1% | 18·9% | 19·6% | 29·9% | 30·2% |
|  | WEST BERKSHIRE | 19·6% | 19·6% | 30·1% | 30·2% | 17·9% | 17·9% | 27·8% | 28·1% |
|  | WEST SUSSEX COUNTY | 21·7% | 21·9% | 30·4% | 30·7% | 19·6% | 19·8% | 26·4% | 26·6% |
|  | WIGAN DISTRICT | 21·4% | 21·4% | 33·0% | 33·0% | 21·9% | 21·9% | 30·7% | 30·7% |
|  | WILTSHIRE | 23·7% | 23·4% | 29·5% | 29·4% | 18·8% | 18·7% | 29·2% | 29·2% |
|  | WINDSOR AND MAIDENHEAD | 15·8% | 17·4% | 25·7% | 27·6% | 16·7% | 17·5% | 29·9% | 30·9% |
|  | WIRRAL DISTRICT | 22·1% | 22·2% | 33·2% | 33·3% | 22·5% | 22·6% | 33·1% | 33·2% |
|  | WOKINGHAM | 17·9% | 17·9% | 27·6% | 28·6% | 15·1% | 15·1% | 24·8% | 25·1% |
|  | WORCESTERSHIRE COUNTY | 24·8% | 25·3% | 33·1% | 33·4% | 21·0% | 21·3% | 30·7% | 31·1% |
|  | YORK | 22·0% | 22·1% | 31·3% | 31·3% | 20·4% | 20·4% | 29·9% | 29·9% |

* Potential Data Quality issues in these areas

** Areas with <1000 individuals

SUPPLEMENTARY TABLE 3: LOCAL AUTHORITY RANKINGS BASED ON OVERWEIGHT-OBESITY BEFORE AND AFTER BMI ADJUSTMENT BY AGE AND SEX IN THE NATIONAL CHILD MEASUREMENT PROGRAMME (2012-13)

| **Area** | **4-5 Years old** | | | |  | **10-11 Years old** | | | |
| --- | --- | --- | --- | --- | --- | --- | --- | --- | --- |
|  | **Boys** | | **Girls** | |  | **Boys** | | **Girls** | |
|  | **Overweight-Obese** | | **Overweight-Obese** | |  | **Overweight-Obese** | | **Overweight-Obese** | |
|  | **Before** | **After** | **Before** | **After** |  | **Before** | **After** | **Before** | **After** |
| BARKING AND DAGENHAM (LONDON BORO) | 8 | 81 | 25 | 84 |  | 17 | 23 | 6 | 15 |
| BARNET (LONDON BORO) | 26 | 66 | 66 | 88 |  | 41 | 52 | 114 | 117 |
| BARNSLEY DISTRICT | 80 | 88 | 29 | 41 |  | 55 | 60 | 41 | 49 |
| BATH AND NORTH EAST SOMERSET | 47 | 62 | 44 | 51 |  | 144 | 144 | 143 | 144 |
| BEDFORD | 46 | 25 | 32 | 23 |  | 53 | 41 | 66 | 67 |
| BEXLEY (LONDON BORO) | 14 | 52 | 2 | 20 |  | 11 | 14 | 13 | 13 |
| BIRMINGHAM DISTRICT | 75 | 13 | 34 | 4 |  | 21 | 7 | 25 | 8 |
| BLACKBURN WITH DARWEN | 96 | 11 | 89 | 3 |  | 110 | 39 | 101 | 40 |
| BLACKPOOL | 13 | 18 | 9 | 17 |  | 42 | 44 | 63 | 68 |
| BOLTON DISTRICT | 128 | 59 | 127 | 74 |  | 50 | 29 | 42 | 27 |
| BOURNEMOUTH | 86 | 75 | 100 | 89 |  | 114 | 113 | 87 | 91 |
| BRACKNELL FOREST | 141 | 140 | 103 | 101 |  | 130 | 135 | 145 | 145 |
| BRADFORD DISTRICT | 87 | 3 | 99 | 7 |  | 40 | 12 | 61 | 22 |
| BRENT (LONDON BORO) | 98 | 95 | 56 | 77 |  | 14 | 24 | 10 | 18 |
| BROMLEY (LONDON BORO) | 91 | 129 | 115 | 136 |  | 94 | 111 | 102 | 113 |
| BUCKINGHAMSHIRE COUNTY | 136 | 118 | 133 | 100 |  | 133 | 128 | 137 | 134 |
| BURY DISTRICT | 114 | 58 | 138 | 129 |  | 72 | 68 | 76 | 63 |
| CALDERDALE DISTRICT | 117 | 41 | 77 | 31 |  | 104 | 81 | 125 | 116 |
| CAMBRIDGESHIRE COUNTY | 121 | 105 | 129 | 115 |  | 138 | 137 | 123 | 123 |
| CAMDEN (LONDON BORO) | 102 | 74 | 119 | 126 |  | 47 | 34 | 46 | 53 |
| CENTRAL BEDFORDSHIRE | 133 | 131 | 128 | 123 |  | 139 | 140 | 128 | 130 |
| CHESHIRE EAST | 113 | 108 | 118 | 109 |  | 125 | 124 | 126 | 126 |
| CHESHIRE WEST AND CHESTER | 51 | 55 | 26 | 33 |  | 120 | 120 | 85 | 86 |
| CITY OF WESTMINSTER (LONDON BORO) | 20 | 42 | 84 | 92 |  | 1 | 3 | 79 | 90 |
| CITY OF BRISTOL | 109 | 114 | 61 | 67 |  | 59 | 59 | 75 | 82 |
| CITY OF DERBY | 71 | 40 | 23 | 12 |  | 108 | 84 | 57 | 36 |
| CITY OF KINGSTON UPON HULL | 30 | 37 | 97 | 86 |  | 46 | 43 | 30 | 31 |
| CITY OF LEICESTER | 123 | 44 | 43 | 10 |  | 49 | 21 | 67 | 46 |
| CITY OF NOTTINGHAM | 76 | 89 | 42 | 53 |  | 66 | 53 | 40 | 39 |
| CITY OF PETERBOROUGH | 29 | 8 | 68 | 42 |  | 64 | 65 | 69 | 71 |
| CITY OF PLYMOUTH | 18 | 27 | 33 | 44 |  | 82 | 89 | 111 | 108 |
| CITY OF PORTSMOUTH | 48 | 63 | 19 | 32 |  | 34 | 35 | 65 | 70 |
| CITY OF SOUTHAMPTON | 68 | 50 | 83 | 66 |  | 90 | 94 | 34 | 33 |
| CITY OF STOKE-ON-TRENT | 41 | 23 | 21 | 16 |  | 38 | 30 | 8 | 5 |
| CITY OF WOLVERHAMPTON DISTRICT | 2 | 2 | 10 | 13 |  | 6 | 6 | 11 | 7 |
| CORNWALL | 21 | 30 | 49 | 55 |  | 112 | 112 | 116 | 115 |
| COUNTY DURHAM | 93 | 98 | 79 | 81 |  | 43 | 46 | 38 | 44 |
| COUNTY OF HEREFORDSHIRE | 124 | 121 | 101 | 98 |  | 124 | 126 | 130 | 132 |
| COVENTRY DISTRICT | 116 | 109 | 114 | 97 |  | 78 | 69 | 44 | 43 |
| CROYDON (LONDON BORO) | 25 | 127 | 57 | 132 |  | 33 | 62 | 12 | 24 |
| CUMBRIA COUNTY | 56 | 65 | 71 | 75 |  | 32 | 33 | 36 | 42 |
| DARLINGTON | 42 | 56 | 110 | 102 |  | 97 | 102 | 55 | 57 |
| DERBYSHIRE COUNTY | 92 | 96 | 113 | 105 |  | 106 | 109 | 86 | 93 |
| DEVON COUNTY | 9 | 15 | 59 | 58 |  | 122 | 123 | 124 | 124 |
| DONCASTER DISTRICT | 95 | 93 | 67 | 69 |  | 56 | 54 | 97 | 95 |
| DORSET COUNTY | 120 | 116 | 135 | 135 |  | 117 | 116 | 140 | 140 |
| DUDLEY DISTRICT | 36 | 26 | 50 | 45 |  | 36 | 32 | 22 | 14 |
| EALING (LONDON BORO) | 85 | 34 | 58 | 71 |  | 12 | 19 | 39 | 48 |
| EAST RIDING OF YORKSHIRE | 132 | 128 | 78 | 80 |  | 81 | 87 | 93 | 96 |
| EAST SUSSEX COUNTY | 110 | 110 | 111 | 112 |  | 115 | 114 | 131 | 131 |
| ENFIELD (LONDON BORO) | 10 | 60 | 8 | 70 |  | 16 | 31 | 15 | 26 |
| ESSEX COUNTY | 115 | 120 | 125 | 119 |  | 113 | 115 | 118 | 119 |
| GATESHEAD DISTRICT | 81 | 90 | 94 | 90 |  | 35 | 37 | 52 | 56 |
| GLOUCESTERSHIRE COUNTY | 16 | 21 | 46 | 56 |  | 93 | 97 | 74 | 78 |
| GREENWICH (LONDON BORO) | 7 | 124 | 6 | 94 |  | 24 | 45 | 9 | 30 |
| HACKNEY (LONDON BORO) | 5 | 53 | 13 | 82 |  | 9 | 25 | 2 | 10 |
| HALTON | 62 | 70 | 4 | 11 |  | 77 | 83 | 7 | 9 |
| HAMMERSMITH AND FULHAM (LONDON BORO) | 15 | 100 | 28 | 117 |  | 28 | 36 | 58 | 94 |
| HAMPSHIRE COUNTY | 129 | 126 | 123 | 116 |  | 134 | 134 | 139 | 139 |
| HARINGEY (LONDON BORO) | 50 | 133 | 81 | 130 |  | 8 | 28 | 16 | 29 |
| HARROW (LONDON BORO) | 103 | 43 | 104 | 48 |  | 31 | 26 | 115 | 110 |
| HARTLEPOOL | 19 | 28 | 15 | 28 |  | 27 | 27 | 19 | 17 |
| HAVERING (LONDON BORO) | 134 | 135 | 65 | 93 |  | 39 | 50 | 68 | 79 |
| HERTFORDSHIRE COUNTY | 130 | 122 | 132 | 122 |  | 127 | 125 | 135 | 136 |
| HILLINGDON (LONDON BORO) | 82 | 73 | 106 | 65 |  | 71 | 56 | 45 | 45 |
| HOUNSLOW (LONDON BORO) | 31 | 12 | 82 | 47 |  | 5 | 4 | 28 | 16 |
| ISLE OF WIGHT | 57 | 68 | 22 | 34 |  | 62 | 63 | 113 | 112 |
| ISLINGTON (LONDON BORO) | 52 | 125 | 55 | 96 |  | 37 | 58 | 31 | 65 |
| KENSINGTON AND CHELSEA (LONDON BORO) | 139 | 145 | 72 | 118 |  | 63 | 92 | 96 | 127 |
| KENT COUNTY | 94 | 92 | 86 | 83 |  | 89 | 98 | 84 | 85 |
| KINGSTON UPON THAMES (LONDON BORO) | 145 | 144 | 144 | 143 |  | 101 | 100 | 132 | 133 |
| KIRKLEES DISTRICT | 99 | 20 | 95 | 30 |  | 99 | 78 | 89 | 69 |
| KNOWSLEY DISTRICT | 23 | 32 | 3 | 8 |  | 18 | 20 | 21 | 21 |
| LAMBETH (LONDON BORO) | 67 | 146 | 35 | 134 |  | 7 | 47 | 17 | 62 |
| LANCASHIRE COUNTY | 39 | 24 | 54 | 40 |  | 85 | 80 | 99 | 87 |
| LEEDS DISTRICT | 74 | 80 | 53 | 49 |  | 74 | 70 | 64 | 61 |
| LEICESTERSHIRE COUNTY | 97 | 82 | 126 | 104 |  | 111 | 107 | 112 | 101 |
| LEWISHAM (LONDON BORO) | 12 | 137 | 41 | 140 |  | 29 | 66 | 14 | 50 |
| LINCOLNSHIRE COUNTY | 17 | 22 | 11 | 21 |  | 79 | 85 | 32 | 34 |
| LIVERPOOL DISTRICT | 1 | 6 | 1 | 6 |  | 13 | 16 | 18 | 20 |
| LUTON | 60 | 7 | 91 | 14 |  | 30 | 9 | 29 | 11 |
| MANCHESTER DISTRICT | 11 | 16 | 18 | 27 |  | 25 | 22 | 5 | 6 |
| MEDWAY | 55 | 61 | 51 | 57 |  | 86 | 79 | 80 | 83 |
| MERTON (LONDON BORO) | 104 | 106 | 107 | 133 |  | 54 | 55 | 56 | 72 |
| MILTON KEYNES | 65 | 99 | 90 | 78 |  | 88 | 93 | 95 | 109 |
| NEWCASTLE UPON TYNE DISTRICT | 3 | 4 | 24 | 18 |  | 20 | 11 | 37 | 32 |
| NEWHAM (LONDON BORO) | 27 | 17 | 17 | 5 |  | 4 | 2 | 4 | 2 |
| NORFOLK COUNTY | 35 | 47 | 76 | 79 |  | 92 | 101 | 100 | 100 |
| NORTHAMPTONSHIRE COUNTY | 84 | 97 | 112 | 113 |  | 91 | 99 | 81 | 84 |
| NORTHUMBERLAND | 69 | 76 | 62 | 63 |  | 100 | 106 | 121 | 121 |
| NORTH EAST LINCOLNSHIRE | 45 | 57 | 63 | 64 |  | 80 | 86 | 54 | 59 |
| NORTH LINCOLNSHIRE | 32 | 35 | 12 | 19 |  | 58 | 51 | 98 | 89 |
| NORTH SOMERSET | 22 | 29 | 40 | 50 |  | 116 | 117 | 105 | 106 |
| NORTH TYNESIDE DISTRICT | 4 | 10 | 20 | 24 |  | 60 | 61 | 47 | 51 |
| NORTH YORKSHIRE COUNTY | 88 | 87 | 116 | 110 |  | 140 | 139 | 129 | 129 |
| NOTTINGHAMSHIRE COUNTY | 100 | 91 | 96 | 91 |  | 107 | 108 | 119 | 114 |
| OLDHAM DISTRICT | 38 | 5 | 39 | 2 |  | 57 | 38 | 78 | 66 |
| OXFORDSHIRE COUNTY | 135 | 132 | 139 | 141 |  | 129 | 132 | 134 | 135 |
| POOLE | 63 | 67 | 47 | 52 |  | 141 | 141 | 136 | 137 |
| READING | 77 | 84 | 87 | 85 |  | 98 | 91 | 27 | 25 |
| REDBRIDGE (LONDON BORO) | 111 | 9 | 121 | 29 |  | 23 | 5 | 71 | 38 |
| RICHMOND UPON THAMES (LONDON BORO) | 142 | 138 | 146 | 146 |  | 132 | 133 | 146 | 146 |
| ROCHDALE DISTRICT | 101 | 19 | 73 | 35 |  | 96 | 75 | 50 | 23 |
| ROTHERHAM DISTRICT | 70 | 45 | 88 | 60 |  | 51 | 42 | 51 | 47 |
| SALFORD DISTRICT | 66 | 83 | 52 | 59 |  | 61 | 72 | 26 | 28 |
| SANDWELL DISTRICT | 105 | 33 | 69 | 36 |  | 15 | 8 | 3 | 4 |
| SEFTON DISTRICT | 28 | 36 | 70 | 73 |  | 67 | 73 | 33 | 35 |
| SHEFFIELD DISTRICT | 131 | 107 | 134 | 121 |  | 68 | 64 | 72 | 74 |
| SHROPSHIRE | 58 | 69 | 105 | 99 |  | 84 | 90 | 90 | 97 |
| SLOUGH | 127 | 94 | 37 | 9 |  | 45 | 10 | 83 | 55 |
| SOLIHULL DISTRICT | 122 | 85 | 140 | 139 |  | 137 | 129 | 133 | 125 |
| SOMERSET COUNTY | 34 | 46 | 64 | 61 |  | 135 | 136 | 122 | 122 |
| SOUTHEND-ON-SEA | 44 | 64 | 74 | 72 |  | 95 | 96 | 104 | 103 |
| SOUTHWARK (LONDON BORO) | 6 | 130 | 7 | 124 |  | 3 | 18 | 1 | 1 |
| SOUTH GLOUCESTERSHIRE | 144 | 142 | 143 | 144 |  | 126 | 130 | 110 | 105 |
| SOUTH TYNESIDE DISTRICT | 83 | 79 | 5 | 15 |  | 19 | 15 | 49 | 41 |
| STAFFORDSHIRE COUNTY | 54 | 51 | 30 | 37 |  | 70 | 74 | 73 | 76 |
| STOCKPORT DISTRICT | 59 | 48 | 120 | 106 |  | 123 | 122 | 82 | 77 |
| STOCKTON-ON-TEES | 125 | 123 | 16 | 25 |  | 65 | 67 | 48 | 52 |
| ST HELENS DISTRICT | 40 | 49 | 14 | 22 |  | 48 | 48 | 35 | 37 |
| SUFFOLK COUNTY | 107 | 101 | 108 | 103 |  | 118 | 119 | 120 | 120 |
| SUNDERLAND DISTRICT | 24 | 31 | 45 | 46 |  | 75 | 76 | 23 | 19 |
| SURREY COUNTY | 140 | 136 | 142 | 142 |  | 142 | 143 | 142 | 141 |
| SUTTON (LONDON BORO) | 118 | 119 | 136 | 128 |  | 69 | 57 | 103 | 99 |
| SWINDON | 73 | 71 | 36 | 39 |  | 73 | 77 | 88 | 81 |
| TAMESIDE DISTRICT | 79 | 86 | 31 | 43 |  | 83 | 88 | 70 | 75 |
| TELFORD AND WREKIN | 37 | 38 | 27 | 26 |  | 52 | 49 | 60 | 58 |
| THE CITY OF BRIGHTON AND HOVE | 90 | 102 | 109 | 107 |  | 143 | 146 | 141 | 142 |
| THURROCK | 61 | 117 | 102 | 125 |  | 44 | 71 | 53 | 73 |
| TOWER HAMLETS (LONDON BORO) | 49 | 1 | 38 | 1 |  | 2 | 1 | 20 | 3 |
| TRAFFORD DISTRICT | 138 | 139 | 124 | 120 |  | 87 | 95 | 77 | 80 |
| WAKEFIELD DISTRICT | 53 | 54 | 80 | 68 |  | 76 | 82 | 59 | 54 |
| WALSALL DISTRICT | 43 | 14 | 75 | 38 |  | 22 | 17 | 24 | 12 |
| WALTHAM FOREST (LONDON BORO) | 89 | 78 | 122 | 111 |  | 10 | 13 | 43 | 60 |
| WANDSWORTH (LONDON BORO) | 72 | 112 | 92 | 131 |  | 26 | 40 | 91 | 111 |
| WARRINGTON | 78 | 72 | 93 | 87 |  | 103 | 103 | 108 | 104 |
| WARWICKSHIRE COUNTY | 126 | 113 | 130 | 114 |  | 119 | 118 | 109 | 102 |
| WEST BERKSHIRE | 137 | 134 | 137 | 137 |  | 131 | 131 | 127 | 128 |
| WEST SUSSEX COUNTY | 112 | 111 | 117 | 108 |  | 128 | 127 | 138 | 138 |
| WIGAN DISTRICT | 119 | 115 | 60 | 62 |  | 109 | 110 | 94 | 98 |
| WILTSHIRE | 64 | 77 | 131 | 127 |  | 136 | 138 | 117 | 118 |
| WINDSOR AND MAIDENHEAD | 146 | 143 | 141 | 138 |  | 146 | 145 | 107 | 92 |
| WIRRAL DISTRICT | 106 | 103 | 48 | 54 |  | 102 | 105 | 62 | 64 |
| WOKINGHAM | 143 | 141 | 145 | 145 |  | 145 | 142 | 144 | 143 |
| WORCESTERSHIRE COUNTY | 33 | 39 | 85 | 76 |  | 105 | 104 | 92 | 88 |
| YORK | 108 | 104 | 98 | 95 |  | 121 | 121 | 106 | 107 |

SUPPLEMENTARY TABLE 4: PREVALENCE OF BEING OVERWEIGHT OR OBESE USING NCMP CLINICAL REPORTING THRESHOLDS^1^ BY AGE GROUP AND SEX IN THE NATIONAL CHILD MEASUREMENT PROGRAMME (2012-13)

|  | **Prevalence of Overweight-Obesity using Clinical thresholds^1^, % (95% CI)** | | | | | | | | | |
| --- | --- | --- | --- | --- | --- | --- | --- | --- | --- | --- |
|  | **Before BMI adjustment** | | **After BMI adjustment** | |  | **Before BMI adjustment** | | **After BMI adjustment** | | |
| **Ethnic Group** | **Overweight** | **Obese** | **Overweight** | **Obese** |  | **Overweight** | **Obese** | **Overweight** | **Obese** |  |
|  | **4-5 Year old Boys (N=297,887)** | | | |  | **10-11 Year old Boys (N=248,783)** | | | |  |
| White | 10·0 (9·8 - 10·1) | 4·6 (4·5 - 4·7) |  |  |  | 14·1 (13·9 - 14·2) | 11·2 (11·1 - 11·4) |  |  |  |
| Black - All | 12·8 (12·2 - 13·3) | 9·7 (9·3 - 10·2) | 4·5 (4·2 - 4·9) | 2·8 (2·6 - 3·1) |  | 16·5 (15·8 - 17·2) | 17·5 (16·8 - 18·2) | 13·9 (13·3 - 14·6) | 11·2 (10·6 - 11·8) |  |
| Black African | 13·6 (12·9 - 14·3) | 10·8 (10·2 - 11·4) | 5·2 (4·7 - 5·6) | 3·1 (2·7 - 3·5) |  | 16·9 (15·9 - 17·8) | 17·7 (16·7 - 18·6) | 14·8 (13·9 - 15·7) | 10·7 (9·9 - 11·4) |  |
| Black Caribbean | 10·6 (9·5 - 11·8) | 6·8 (5·9 - 7·8) | 3·1 (2·5 - 3·7) | 1·8 (1·3 - 2·3) |  | 15·8 (14·4 - 17·1) | 18 (16·7 - 19·4) | 12 (10·9 - 13·2) | 12·9 (11·7 - 14·1) |  |
| Black - Other | 12·2 (11·0 - 13·5) | 9·3 (8·2 - 10·4) | 3·9 (3·2 - 4·7) | 3·0 (2·4 - 3·7) |  | 16·7 (15·1 - 18·2) | 16·4 (14·9 - 18) | 14·1 (12·6 - 15·5) | 10·3 (9 - 11·6) |  |
| South Asian - All | 7·0 (6·7 - 7·4) | 7·6 (7·2 - 7·9) | 15·5 (15·0 - 15·9) | 13·4 (13·0 - 13·9) |  | 17·2 (16·7 - 17·8) | 17·3 (16·8 - 17·8) | 20·2 (19·6 - 20·8) | 23·7 (23·1 - 24·3) |  |
| South Asian - Indian | 6·0 (5·5 - 6·6) | 6·2 (5·6 - 6·7) | 13·9 (13·1 - 14·7) | 11·1 (10·4 - 11·9) |  | 17·1 (16·1 - 18·1) | 15·3 (14·4 - 16·2) | 20·3 (19·3 - 21·4) | 21·3 (20·3 - 22·4) |  |
| South Asian - Pakistani | 7·5 (7·0 - 8·0) | 7·9 (7·4 - 8·3) | 16·4 (15·8 - 17·1) | 14·1 (13·5 - 14·8) |  | 16·9 (16·1 - 17·6) | 17·3 (16·5 - 18) | 19·8 (19 - 20·6) | 23·5 (22·6 - 24·3) |  |
| South Asian - Bangladeshi | 7·5 (6·7 - 8·3) | 9·3 (8·4 - 10·1) | 15·6 (14·5 - 16·7) | 15·5 (14·4 - 16·6) |  | 18·2 (17 - 19·5) | 20·4 (19·1 - 21·7) | 21·1 (19·8 - 22·4) | 27·7 (26·3 - 29·1) |  |
| Other Asian | 8·7 (8·0 - 9·4) | 6·9 (6·3 - 7·5) |  |  |  | 18·6 (17·5 - 19·8) | 15·9 (14·8 - 17) |  |  |  |
| Other Ethnicity | 9·9 (9·5 - 10·3) | 6·1 (5·7 - 6·4) |  |  |  | 15·7 (15·1 - 16·3) | 15·1 (14·5 - 15·7) |  |  |  |
| Unknown | 10·1 (9·8 - 10·4) | 5·5 (5·2 - 5·7) |  |  |  | 15·1 (14·8 - 15·5) | 12 (11·7 - 12·3) |  |  |  |
| Overall | 9·9 (9·7 - 10·0) | 5·3 (5·3 - 5·4) | 10·1 (10·0 - 10·2) | 5·5 (5·4 - 5·5) |  | 14·8 (14·6 - 14·9) | 12·4 (12·3 - 12·5) | 14·9 (14·7 - 15) | 12·6 (12·5 - 12·7) |  |
|  | **4-5 Year old Girls (N=285,012)** | | | |  | **10-11 Year old Girls (N=236,579)** | | | |  |
| White | 8·9 (8·8 - 9·1) | 4·1 (4 - 4·2) |  |  |  | 13·8 (13·6 - 14) | 9·1 (8·9 - 9·2) |  |  |  |
| Black - All | 11·8 (11·2 - 12·3) | 9·4 (8·9 - 9·8) | 5·3 (5·0 - 5·7) | 3·1 (2·8 - 3·4) |  | 18·5 (17·8 - 19·3) | 17·5 (16·8 - 18·2) | 15·2 (14·6 - 15·9) | 11·3 (10·7 - 11·8) |  |
| Black African | 12·4 (11·7 - 13·1) | 10 (9·4 - 10·7) | 5·7 (5·2 - 6·2) | 3·3 (2·9 - 3·7) |  | 18·9 (17·9 - 19·9) | 17·4 (16·4 - 18·3) | 15·7 (14·8 - 16·6) | 11 (10·3 - 11·8) |  |
| Black Caribbean | 10·6 (9·4 - 11·7) | 8·6 (7·5 - 9·6) | 4·7 (3·9 - 5·5) | 3 (2·4 - 3·6) |  | 18 (16·6 - 19·4) | 18·7 (17·2 - 20·1) | 15·4 (14·1 - 16·7) | 12·3 (11·1 - 13·5) |  |
| Black - Other | 10·9 (9·7 - 12·1) | 8·1 (7 - 9·1) | 4·8 (4 - 5·7) | 2·5 (1·9 - 3·1) |  | 18·3 (16·7 - 19·9) | 16·1 (14·6 - 17·7) | 13·9 (12·5 - 15·3) | 10·5 (9·2 - 11·8) |  |
| South Asian - All | 7·7 (7·4 - 8·1) | 5·9 (5·6 - 6·3) | 14·2 (13·7 - 14·6) | 10·7 (10·3 - 11·1) |  | 15·3 (14·8 - 15·9) | 11·3 (10·9 - 11·8) | 18·9 (18·3 - 19·4) | 15·9 (15·4 - 16·4) |  |
| South Asian - Indian | 6·5 (5·9 - 7·1) | 4·6 (4·1 - 5·1) | 12 (11·3 - 12·8) | 8·7 (8 - 9·3) |  | 13·9 (13 - 14·8) | 9·1 (8·3 - 9·8) | 18 (17 - 19·1) | 12·8 (11·9 - 13·7) |  |
| South Asian - Pakistani | 8·1 (7·6 - 8·7) | 6·2 (5·8 - 6·7) | 15·2 (14·5 - 15·8) | 11·2 (10·6 - 11·8) |  | 15·4 (14·7 - 16·2) | 12·4 (11·7 - 13) | 19 (18·2 - 19·8) | 17 (16·2 - 17·7) |  |
| South Asian - Bangladeshi | 8·7 (7·8 - 9·6) | 7·6 (6·8 - 8·5) | 15·3 (14·2 - 16·4) | 13 (11·9 - 14) |  | 17·2 (16 - 18·4) | 12 (11 - 13·1) | 19·7 (18·4 - 20·9) | 17·7 (16·5 - 19) |  |
| Other Asian | 7·5 (6·8 - 8·1) | 4·8 (4·3 - 5·4) |  |  |  | 13·3 (12·3 - 14·3) | 8 (7·2 - 8·8) |  |  |  |
| Other Ethnicity | 9 (8·6 - 9·4) | 5·7 (5·3 - 6) |  |  |  | 15·1 (14·4 - 15·7) | 12·6 (12 - 13·2) |  |  |  |
| Unknown | 8·8 (8·5 - 9·1) | 4·8 (4·6 - 5) |  |  |  | 14·1 (13·8 - 14·5) | 9·6 (9·3 - 9·8) |  |  |  |
| Overall | 8·9 (8·8 - 9) | 4·7 (4·6 - 4·8) | 9·1 (9 - 9·2) | 4·8 (4·7 - 4·9) |  | 14·3 (14·1 - 14·4) | 9·9 (9·8 - 10) | 14·4 (14·2 - 14·5) | 10 (9·9 - 10·1) |  |

^1^ Clinical thresholds: Overweight: ≥91^st^ centile & Obese: ≥98^th^ centile
